# Quantitative Analysis of Protein–Protein Equilibrium Constants in Cellular Environments Using Single-Molecule Localization Microscopy

**DOI:** 10.1021/acs.nanolett.4c04394

**Published:** 2024-10-21

**Authors:** Luis F. Marcano-García, Cecilia Zaza, Olivia P. L. Dalby, Megan D. Joseph, M. Victoria Cappellari, Sabrina Simoncelli, Pedro F. Aramendía

**Affiliations:** † Centro de Investigaciones en Bionanociencias - “Elizabeth Jares-Erijman” (CIBION), CONICET, Godoy Cruz 2390, 1425 Ciudad de Buenos Aires, Argentina; ‡ London Centre for Nanotechnology, University College London, 19 Gordon Street, WC1H 0AH London, United Kingdom; § Department of Chemistry, University College London, 20 Gordon Street, WC1H 0AJ London, United Kingdom

**Keywords:** equilibrium constant, single-molecule localization microscopy, protein−protein interactions, DNA-PAINT, T cells

## Abstract

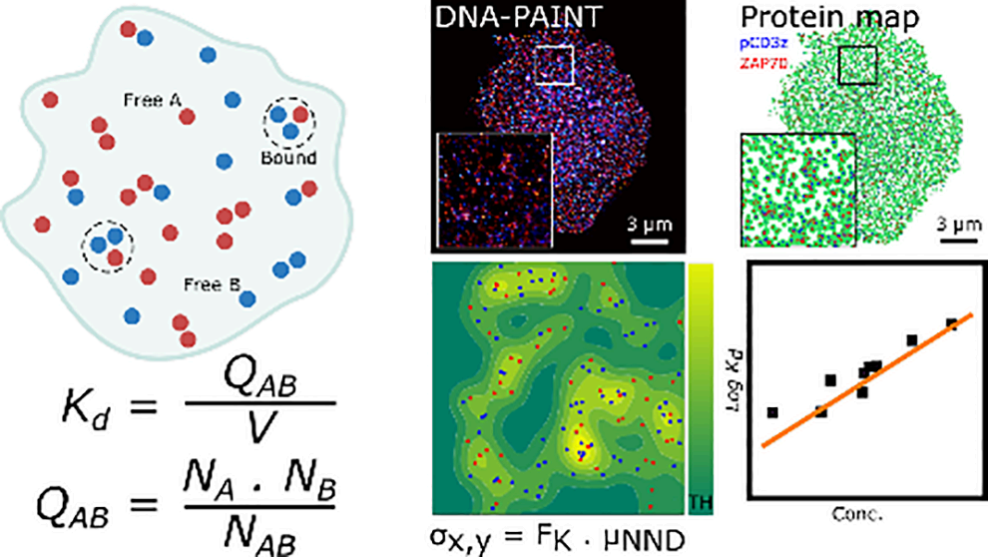

Current methods for determining equilibrium constants often operate in three-dimensional environments, which may not accurately reflect interactions with membrane-bound proteins. With our technique, based on single-molecule localization microscopy (SMLM), we directly determine protein–protein association (*K*_a_) and dissociation (*K*_d_) constants in cellular environments by quantifying associated and isolated molecules and their interaction area. We introduce Kernel Surface Density (ks-density,) a novel method for determining the accessible area for interacting molecules, eliminating the need for user-defined parameters. Simulation studies validate our method’s accuracy across various density and affinity conditions. Applying this technique to T cell signaling proteins, we determine the 2D association constant of T cell receptors (TCRs) in resting cells and the pseudo-3D dissociation constant of pZAP70 molecules from phosphorylated intracellular tyrosine-based activation motifs on the TCR-CD3 complex. We address challenges of multiple detection and molecular labeling efficiency. This method enhances our understanding of protein interactions in cellular environments, advancing our knowledge of complex biological processes.

The thermodynamics of protein interactions within cells play an essential role in several biological processes, such as enzyme catalysis,^1^ cellular structure,^2,3^ or immune responses.^4^ However, determining association constants, *K*_a_, in physiological environments is complex. Molecular association depends on concentration, governed by the thermodynamic chemical potential of each species, which in turn depends on their density and interactions, determined by the molecular nature and environment.^5^ Consequently, the degree of association in cellular environments may differ significantly from that in simple solutions.^6^ Still, many experimental methods to study association constants rely on having free proteins in solutions.^7^ Techniques like NMR,^8^ thermophoresis,^9^ stopped-flow spectrofluorimetry,^5^ analytical ultracentrifugation,^10^ and calorimetric methods (differential scanning calorimetry or isothermal titration calorimetry^11−13^) are reliable but require high concentrations of samples and measure protein interactions in 3D. However, *in vivo*, several protein interactions occur in two phases (membrane and cytosol) or in 2D (membrane processes), leading to the difference in dimension for the association constant (volume in 3D to area in 2D).^14^ Surface plasmon resonance (SPR), which involves immobilizing one of the two proteins on a surface while the other remains in solution, is widely used for studying interactions in two-phase systems,^15^ but immobilization of proteins on a surface can potentially alter their conformation and affect interaction dynamics. On the other hand, to retrieve 2D association constants, mechanical-based methods, including micropipette adhesion frequency,^16^ and fluorescence-based imaging methods,^16,17^ involving fluorescence recovery after photobleaching (FRAP)^17^ and single-molecule fluorescence resonance energy transfer (FRET),^6^ were utilized, particularly, for ligand–receptor interactions. These approaches revealed a sharp contrast between 2D and 3D kinetic and thermodynamic information,^6^ emphasizing the need for a simple method to determine 2D association constants of proteins within their cellular environment, beyond membrane-bound receptor–ligand interactions.

In this paper, we present an analysis technique based on single-molecule localization microscopy (SMLM) to determine protein–protein association, *K*_a_, or dissociation, *K*_d_, constants in their cellular environments. SMLM uses isolated fluorescent molecules to create super-resolved images with nanometer precision. Counting associated and isolated molecules can render a quantitative parameter to evaluate the strength of the interaction (Figure 1a). We showed an example based on this approach to determine the *K*_a_ of complementary DNA sequences.^18^ Now, we extend the analysis to membrane protein association and dissociation of membrane complexes, releasing one component into the cytosol.

**Figure 1:**
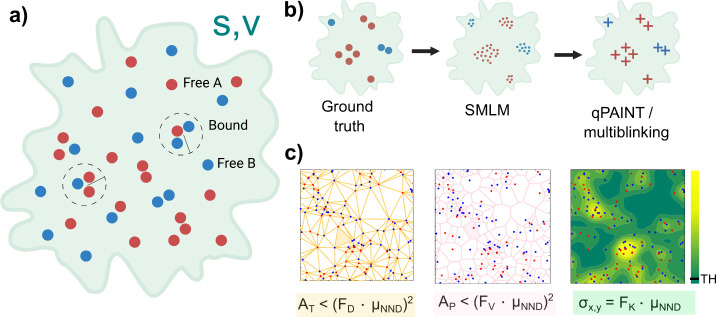
Workflow for determining association constants from SMLM data. (a) SMLM imaging provides information that can be used to correlate localizations and count associated and isolated molecules from a proximity criterion. With this criterion, and a reference distribution, we can render a quantitative parameter to evaluate the strength of the association. Here, red circles represent an example species “A” and blue circles represent an example species “B” at different positions within a cell. Here both the free and bound species can be imaged with SMLM. Concentrations of both species A and B can be expressed per unit surface or volume (S,V). Radial solid line within the dashed circle indicates the proximity criterion utilized to consider species as either free or associated. (b) Schematic of SMLM imaging comparing the ground truth data with SMLM-imaged data followed by quantitative (qPAINT) or multiblinking analysis. Showing example species “A” in red circles, and “B” in blue circles, with their original ground truth positions, results from SMLM data and final protein map from analysis. SMLM renders clusters of single-molecule localizations around the ground-truth position of the target proteins. To accurately determine the number of associated and isolated molecules, SMLM data sets must be corrected for multiblinking or subjected to qPAINT analysis, depending on the imaging modality. (c) To compute the space occupied by the interacting species, we consider three different methods that use molecular coordinates to estimate the pattern containing the molecules: Delaunay (left) and Voronoi (middle) tessellations and a kernel surface density function, ks-density (right). For the tessellations, the acceptance area of the triangle (*A*_T_) or the polygon (*A*_P_) is set based on the average of the first nearest neighbor distance (μ_NND_) weighted by suitable scaling factors, *F*_D_ and *F*_V_, respectively. For ks-density, two parameters are considered, the Gaussian point dispersion (σ_*x*,*y*_), set by μ_NND_ weighted by scaling factor (*F*_K_) and the rejection threshold (TH) as described in the Methods (Supporting Information).

Ideally, in a 1:1 interaction of two species A and B to form a complex AB: *K*_a_ = [AB]/([A]·[B]), concentrations can be expressed per unit surface or volume. However, several factors must be considered when computing concentrations from SMLM experiments: (1) multiple detection of the same molecule can render artificial overcounting or clustering, due to emission blinking in PALM^19^ or STORM,^20^ or repetitive binding–unbinding cycles in DNA-PAINT^21^ (Figure 1b); (2) discrepancy between counted molecules and the real value responsible for the chemical potential, due to labeling efficiency and fluorescence detectability; (3) localization precision, still bigger than the molecular size, requiring a proximity criterion and reference distribution; (4) the area or volume containing the molecules as association–dissociation depends on molecular density. The multiple counting problem can be diminished by correcting based on the SMLM technique. For PALM and STORM, this requires careful calibration and knowledge of the complex photophysical properties of the fluorophores to correct for multiblinking.^22^ In DNA-PAINT, calibration depends on the reversible binding kinetics between docking and imager strands, modality known as qPAINT^23−27^ (Figure 1b). The second issue can be accounted for in a 1:1 association or dissociation reaction if measurements are performed as a function of the total molecular density. The actual value of the *K*_a_ or *K*_d_ can be obtained by extrapolation to infinite dilution (Supplementary Note 1).^18^ The correlation problem can be treated by establishing a proximity distance and using the random distribution as a reference of nonassociated partners. Finally, the magnitude of the area or volume containing the molecules can be determined by methods based on the molecular density. They include Delaunay^28,29^ (Figure 1c, left) and Voronoi^30,31^ (Figure 1c, middle) tessellations and kernel surface density function, ks-density^32,33^ (Figure 1c, right). These are described within the Methods (Supporting Information). All three methods distinguish void or isolated areas from the core region containing the majority of molecules and provide quantitative measurement of this area. When needed, the volume can be determined by multiplying the core area by the illumination volume, Total Internal Reflexion Fluorescence (TIRF) depth, which ranges from 100 to 250 nm.

The capability of computing 2D association constants from SMLM data sets was tested by simulating 2D molecular distributions of two species, at different molecular densities (from 120 to 400 μm^–2^) and association affinities (as measured by log *K*_a_, from −3 to 0 on a μm^–2^ scale), in environments mimicking cellular compartments (Figure 2a). Figure 2b presents the molecular localizations across four different combinations of extreme density and affinity. From the molecular coordinates, we used Delaunay and Voronoi tessellations, and ks-density, to recover the patterns containing the localizations. Figure 2c displays the tessellation graphs and the color scale representation of the ks-density function (top) and compares the original pattern (Figure 2a) with those recovered (bottom) using Delaunay (Figure 2c, left), Voronoi (Figure 2c, middle), and ks-density (Figure 2c, right). This example has been computed for a total molecular density of 160 μm^–2^ and log *K*_a_ = −2.0. Additional recovered patterns for simulations performed at other molecular densities and affinity are presented in Supplementary Figure 1. Finally, Figure 2d shows the difference between the computed and input values of −Δ*G*°/*RT* = log *K*_a_ as a function of the density and affinity. We present a summary of the implementation of each pattern analysis algorithm, along with the optimization of their relevant parameters. More details can be found in the Supporting Information.

**Figure 2:**
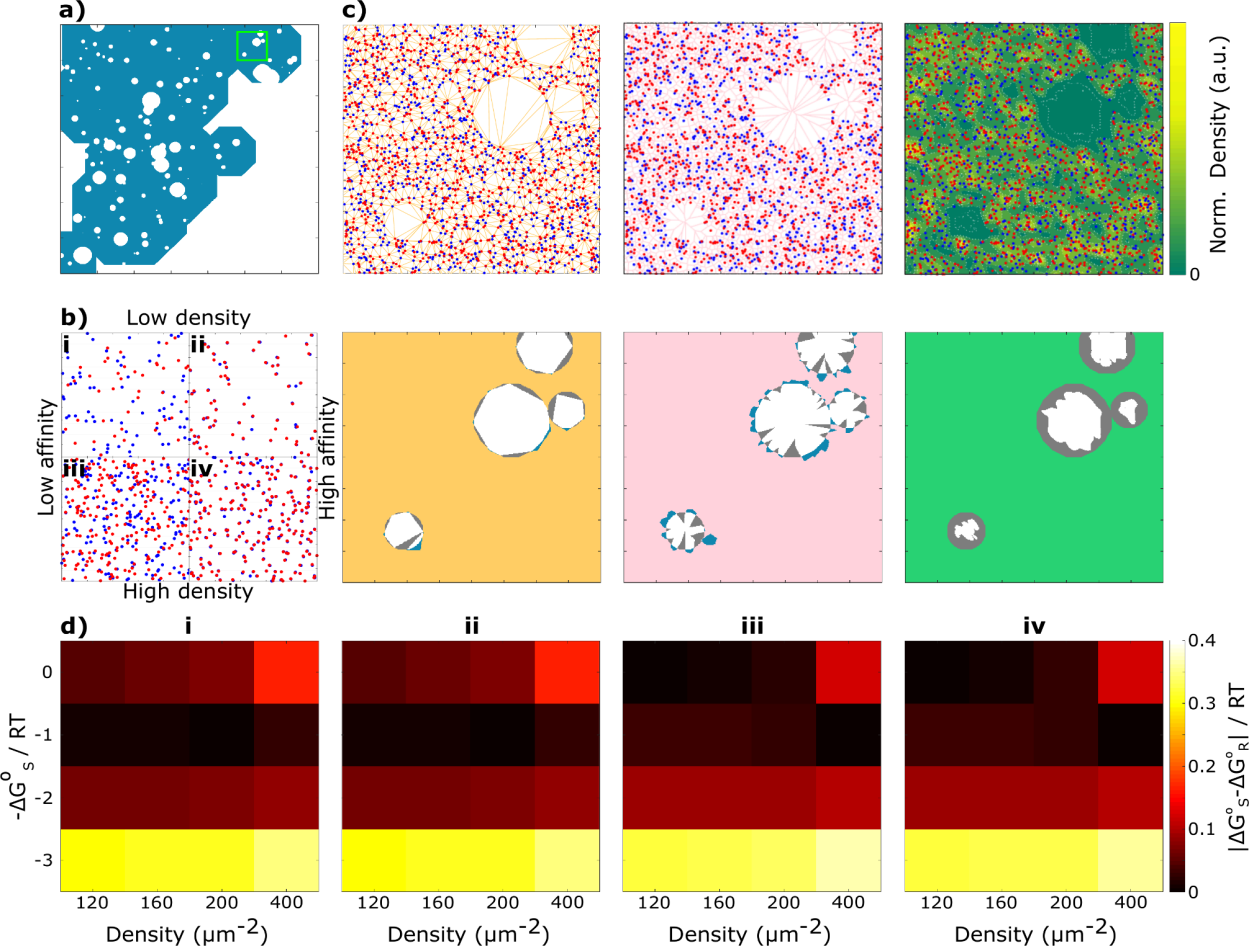
Method performance computed from 2D simulations of interacting molecular species in environments that mimic cell shapes. (a) Example of a simulated pattern of 692 μm^2^ in a 35 × 35 μm^2^ frame. (b) Simulations under different extreme conditions on a 1 × 1 μm^2^ region: (*i*) density = 120 μm^–2^ and log *K*_a_ = −3, (*ii*) density = 120 μm^–2^ and log *K*_a_ = 0, (*iii*) density = 400 μm^–2^ and log *K*_a_ = −3, and (*iv*) density = 400 μm^–2^ and log *K*_a_ = 0. (c) The smaller region of 4 × 4 μm^2^ indicated in panel (a) with a total density of 160 μm^–2^. Here, there are equal amounts of each species with a log *K*_a_ = −2 and no localizations are out of the pattern, with the following methods: Top, Left: Delaunay tessellation. Top, Middle: Voronoi tessellation. Top, Right: ks-density with Gaussian expansion of *F*_K_ = 1. Bottom: Recovered patterns with the respective expansion factor that are compared to the original one (FP, gray; FN, sky-blue; TN, white; and TP, yellow, pink, and green to Delaunay, Voronoi tessellation, and ks-density methods, respectively). Left: Delaunay tessellation method considering all triangles with area: *A*_T_ ≤ (3·μ_NND_),^2^ F1 = 0.99. Middle: Voronoi tessellation method considering all polygons with area: *A*_P_ ≤ (3·μ_NND_),^2^ F1 = 0.98. Right: ks-density method with σ_*x,y*_ = 1·μ_NND_ and TH given by equation in the Methods, F1 = 0.87. (d) Difference in -Δ*G*°/*RT* between the recovered value (R subindex) and the value used in the simulation (S subindex), in patterns recovered with F1 ≥ 0.86 for the three methods: (*i*) Delaunay tessellation method with *F*_D_ = 3. (*ii*) Voronoi tessellation method with *F*_V_ = 3. ks-density function method with *F*_K_ = 1 and (*iii*) TH = 1.5/(·σ_*x*,*y*_·*N*_T_); (*iv*) TH = 2.5/(·σ_*x*,*y*_·*N*_T_). All simulations are described in the Methods (Supporting Information).

The key reference to parametrize all three methods is the average of the distance to the first nearest neighbor (NND) of the total distribution, μ_NND_, an unbiased parameter easily accessible from the raw data. Based on μ_NND_, an expansion factor (*F*_D_, *F*_V_, or *F*_K_ for Delaunay, Voronoi, or ks-density, respectively) was used as a parameter to optimize the coincidence between the simulated and recovered area. F1 score of the confusion matrix was used as an optimization criterion. The results are summarized in Supplementary Table 1 and Supplementary Figure 2.

After recovering the pattern using each method, molecules included were individualized, and their locations were used to identify associated and isolated ones. As described (Methods, Supporting Information), the most suitable proximity limit to consider association in these simulations was established as 15 nm, by considering the average uncertainty in the location of pairs and searching the coincidence of the computed *K*_a_ value with the input value within a range of 0.3 logarithmic units (Supplementary Figure 3). Across the simulated density and affinity ranges, Δ*G*°/*RT* could be recovered with a deviation of ±0.3 with respect to the computed value, using any of the three methods, except at the lowest affinity and highest density, where deviations scale up to ±0.4. A difference of ±0.3 in Δ*G*°/*RT* at 25 °C is equivalent to ±743 J/mol in Δ*G*°, a very low energy difference, supporting the whole procedure. Although *K*_a_ is unknown, the parameters can be optimized through iteration. Notably, the ks-density shows the least sensitivity to parameter changes (Supplementary Table 1 and Supplementary Figure 2) and requires less computational time than tessellation methods. Keeping this in mind, in what follows we show results obtained using ks-density to stress the applicability of this method. As mentioned above, the extension of this procedure to 3D concentrations involves simply multiplying the area by the observation depth in TIRF.

To highlight the method’s potential for determining 2D association constants of biologically relevant partners in situ, we applied the validated analysis to examine the association of T cell receptors (TCR) in the context of T cell signaling. T cells play a central role in pathogen elimination and tumor surveillance by identifying, as quickly and precisely as possible, harmful antigens displayed on major histocompatibility complexes (MHCs) expressed on the surface of antigen-presenting cells (APCs). To achieve antigen sensitivity and specificity, TCRs associate to form preactivation nanoscale clusters, which then increase in size and number upon T cell activation.^34−37^ Here, we quantified the 2D association constants between TCRs located in the membrane of nonactivated T cells from SMLM data.

Figure 3a shows a representative super-resolved image of TCRs in nonactivated Jurkat T cells obtained via DNA-PAINT imaging under TIRF excitation. For imaging, TCRs were labeled with primary antibodies, targeting the CD3ζ subunit, chemically coupled to orthogonal docking sequences featuring a repetitive (AC)n sequence^38^ (Figure 3b), resulting in 6 nm localization precision (Supplementary Figure 4).^39^ To quantify antibody-labeled CD3ζ proteins, we subjected the DNA-PAINT data to qPAINT analysis (Methods, Supporting Information). Figure 3c shows the histogram of the inverse of the measured dark times, known as the qPAINT indexes (QPI), which is fitted to the sum of two Gaussian functions with peaks located at multiples of a QPI value of 0.017 s^–1^ for the CD3ζ docking-imager pair. This value, in combination with a distance-based algorithm, was used to recover an accurate quantitative map of the nanoscale distribution of antibody-labeled CD3ζ proteins in nonactivated Jurkat T cells. Figure 3d shows the cluster size distribution. With this information at hand and using the method presented in this work, we determined the molecular pattern of the space occupied by the TCRs. Figure 3e shows the recovered pattern using the ks-density method in green, with the receptor locations in purple. The whole-cell analysis of the first NND of CD3ζ proteins for the same Jurkat T cell is displayed in Figure 3f. In this same plot, it is compared to the first NNDs histogram of the random distribution as a reference of nonassociated partners (i.e., corresponding to the case of complete spatial randomness (CSR), computed at the experimentally measured density), demonstrating the intrinsic association between TCRs in nonactivated Jurkat T cells.

**Figure 3:**
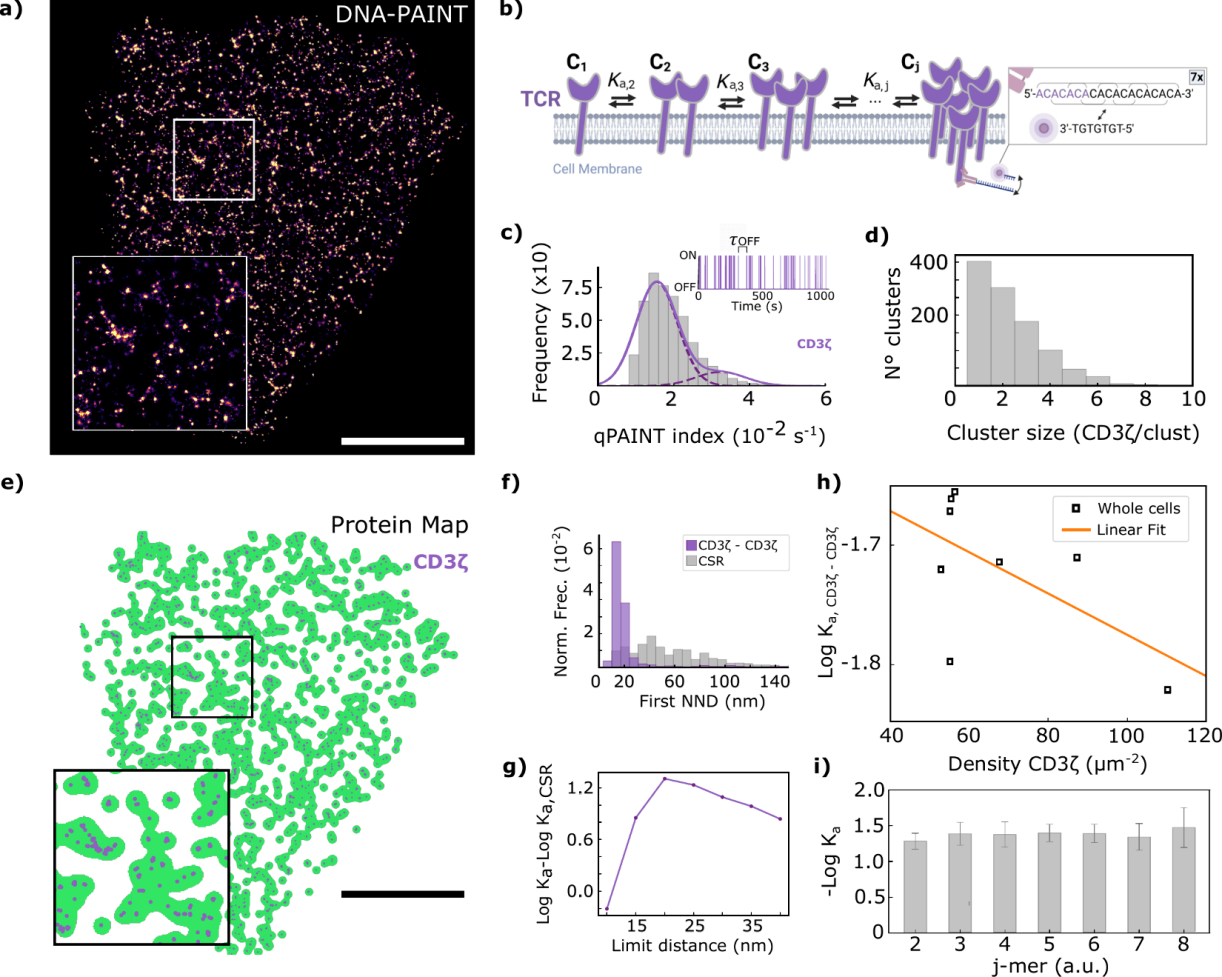
Association analysis of T cell receptors (TCR) in resting Jurkat T cells. (a) Super-resolution DNA-PAINT image of CD3ζ proteins in a representative nonactivated Jurkat T cell. (b) Schematic representation of the sequential association of TCRs characterized by the corresponding association constant *K*_a,j_. Inset shows DNA docking and imager strand sequences displaying the 7x repeat binding motif. (c) Histogram of qPAINT indexes, defined as (τ_OFF_)^−1^, for CD3ζ single molecule localization clusters. The fit to a sum of two Gaussian functions is shown as solid lines, whereas each component is shown as a dashed line. Inset shows blinking kinetics of an example single molecule localization cluster of CD3ζ with indicated dark time (τ_OFF_). (d) Distribution of CD3ζ cluster sizes from qPAINT analysis from the example cell depicted in panel a. (e) Surface area of Jurkat T cell CD3ζ distribution as calculated by ks-density (green). Purple points represent the CD3ζ protein distribution. (f) Whole-cell analysis of first nearest neighbor distances (NNDs) of CD3ζ (purple). The histogram of NNDs for complete spatial randomness (CSR) is represented in gray. (g) Difference between log *K*_a_ for CD3ζ and a random distribution as a function of the proximity limit threshold utilized to consider proteins as associated pairs for the cell presented in Figure 3a. (h) log *K*_a_ as a function of total molecular density with a linear fit used to extrapolate the value of log *K*_a,limit_. Each data set (squares) in this graph corresponds to the analysis from whole-cell images. (i) log *K*_a,*j*_ for the stepwise formation of clusters of *j* number of CD3ζ proteins from the association of a monomer to a cluster of *j* – 1 proteins. Values are the average for the eight cells shown in Supplementary Figure 5. Zoom area in panels (a) and (e) is 2 × 2 μm^2^. Scale bar represents 3 μm.

To determine the proximity threshold for identifying free vs bound proteins, we calculated log *K*_a_ as a function of the threshold and compared it to CSR at the same density (Figure 3g and Supplementary Figure 5). The greatest difference between experimental *K*_a_ and CSR lies in the 20–25 nm range, matching the maximum of the first NNDs histogram. This result is consistent across three independent experiments and data from 8 cells (Supplementary Figure 5). A 25 nm value was used as the proximity limit. The criterion used here differs from the one used in the simulations because the average localization uncertainty of the proteins in qPAINT is not as precisely known as in the simulations, and the true value of *K*_a_ is unknown. On the other hand, the comparison with CSR can always be performed for experimental data.

As a result of incomplete labeling detection of the proteins, there should be a slight dependence of the computed *K*_a_ on molecular density. The corrected value of this parameter can be obtained by extrapolation to infinite dilution. Figure 3h shows the values of *K*_a_ as a function of molecular density for the 1:1 association of CD3ζ in whole cells. This plot represents the expected tendency with density due to incomplete labeling detection expressed in eq S8 of Supplementary Note 1. The extrapolated value is *K*_a_ = (25 ± 10) × 10^–3^ on the μm^2^ scale.

Cluster formation can be visualized as a stepwise process involving the association–dissociation of one protein at a time, as shown in Figure 3b. At equilibrium, this results in a distribution of the number of proteins per cluster. The distribution of cluster size is shown in Figure 3d and Supplementary Figure 6. From this distribution, we can calculate the equilibrium constant for each step as *K*_a,*j*_ = [*N*_*j*_/(*N*_*j*–1_*N*_1_)]*A*, where *j* represents the number of proteins in each cluster, *N*_1_ being the monomer and *A* the area. For a *j* between 2 and 8, the values of log *K*_a,*j*_ are shown in Supplementary Table 2. They are all around log *K*_a,2–8_ = −1.4 on the μm^2^ scale (Figure 3i). This is not the prediction of the simplest Poisson type association, which renders decreasing association constants with an increasing number of proteins, as a consequence of size independent association and a size increasing dissociation rate (eq S12). If we agree that association of a monomer is mainly dependent on its diffusion ability independent of cluster size, then we can conclude that results show an increasing residence time per monomer with cluster size, pointing to cluster stabilization.

To showcase the method’s ability to compute equilibrium constants in pseudo-3D environments, we next examine the dissociation of phosphorylated protein tyrosine kinase ZAP70 (pZAP70) from phosphorylated intracellular tyrosine-based activation motifs (ITAMs) on the TCR complex (pCD3ζ) in the T cell membrane (Figure 4). This process is significant because the release of active ZAP70 (pZAP70) to the cytosol is believed to enable the phosphorylation of the linker for activation of T cells (LAT) at distant membrane sites and nearby vesicles, contributing to downstream T cell activation.^40,41^ This dissociation process involves species in the membrane (pCD3ζ and pCD3ζ-pZAP70 complex), and the cytosol (pZAP70) and will be evaluated on a molar scale. To determine the *K*_d_ of pZAP70 from pCD3ζ, we first activate T cells with a planar glass-supported lipid bilayer (SLB) functionalized with anti-CD3 and anti-CD28 antibodies to promote TCR engagement. When a T cell comes into contact with the SLB, the TCRs on the cell surface interact with the ligands embedded in the SLB, leading to T cell activation^42−45^ (Figure 4b).

**Figure 4:**
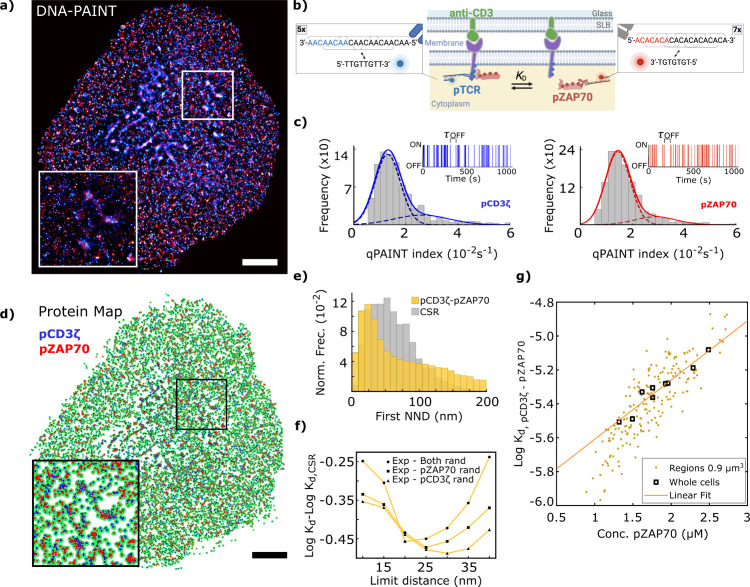
Dissociation analysis of pZAP70 from phosphorylated T cell receptors, pCD3ζ. (a) Super-resolution DNA-PAINT image of pCD3ζ (blue) and pZAP70 (red) proteins in a representative activated Jurkat T cell. (b) Schematic representation of the TCR ζ chains depicting the location of ZAP-70 binding, phosphorylation sites and antibody binding sites for super-resolution DNA-PAINT imaging. Inset shows DNA docking and imager strand sequences displaying the 5x and 7x repeat binding motif. (c). Histogram of qPAINT indexes, defined as (τ_OFF_)^−1^, for pCD3ζ (blue, left) and pZAP70 (red, right) single molecule localization clusters. The fit to a sum of two Gaussian functions is shown as solid lines, whereas each component is shown as a dashed line. Inset shows blinking kinetics of an example single molecule localization cluster of pCD3ζ (blue) and pZAP70 (red) with indicated dark time (τ_OFF_). (d) Surface area (green) of Jurkat T-cell pCD3ζ and pZAP70 distribution as calculated by ks-density. Blue and red points represent pCD3ζ and pZAP70 protein distributions, respectively. (e) Whole-cell analysis of first nearest neighbor distances (NNDs) of pCD3ζ-pZAP70 (top). The histogram of NNDs for CSR is represented in gray. (f) Difference between log *K*_d_ for pCD3ζ-pZAP70 and a random distribution as a function of the proximity limit threshold utilized to consider proteins as associated pairs for the cell presented in Figure 3a. CSR was computed considering three possibilities, either of the two components in its experimental distribution and the other in CSR or both in CSR. (g) log *K*_d_ as a function of pZAP70 concentration with a linear fit used to extrapolate the value of log *K*_d,limit_. Each point in this graph corresponds to values computed in randomly selected 3 × 3 μm^2^ sections of the DNA-PAINT pCD3ζ and pZAP70 Jurkat T cell images. Analysis from whole-cell images is represented with squares. Zoom area in panels (a) and (d) is 4 × 4 μm^2^. Scale bar represents 3 μm.

Figure 4a shows a super-resolved image of pCD3ζ (blue) and pZAP70 (red) proteins in activated Jurkat T cells, obtained using DNA-PAINT imaging under TIRF excitation; localization precision was 10 nm for both pseudocolors (Supplementary Figure 7). DNA-PAINT data were subjected to qPAINT analysis to quantify the number of labeled pCD3ζ and pZAP70 proteins. Figure 4c shows QPI histograms and representative single-molecule ON/OFF time series for clusters in the pCD3ζ and pZAP70 data sets (blue and red, respectively). Figure 4d shows the recovered distribution map of both proteins with their spatial pattern calculated via ks-density. It is noteworthy that control DNA-PAINT imaging of pCD3ζ alone confirms that there is no significant undercounting due to potential steric hindrance in the dual-labeling of pCD3ζ and pZAP70. Quantification revealed 77 ± 2 and 80 ± 5 pCD3ζ proteins per μm^2^ in single- and dual-labeling experiments, respectively.

The first NNDs histogram comparison with a random distribution demonstrates the association between pCD3ζ and pZAP70 (Figure 4e). Analysis of log *K*_d_ as a function of pair distance shows that the proximity threshold for distinguishing free from bound proteins is 20–25 nm (Figure 4f and Supplementary Figure 8), based on data from nine FOVs and 11 cells. For heterospecies analysis, the CSR distribution was computed in three scenarios (each component in the experimental distribution was paired with the other in CSR, and vice versa, or both in CSR), all yielding similar NND distributions. Figure 4g shows the *K*_d_ of pCD3ζ and pZAP70 heterodimers as a function of pZAP70 concentration for whole cells (squares) and 9 μm^2^ and 100 nm depth cell portions comprising at least 1000 protein locations. Both data sets show similar linear correlations, indicating consistent behavior across the whole cell. A linear extrapolation from the whole-cell analysis gives a *K*_d_ value of (1.0 ± 0.4) μM.

If we consider now the sequential model of Taylor et al.,^46^ the analysis of the population distribution of pZAP70 molecules in pCD3ζ clusters of *j* number of members renders the probability that one position is coordinated, *p*_bound,*j*_ (eq S13a). In the Langmuir-type association, this value is a function of *j* and the concentration of pZAP70. The relative distribution of clusters of pCD3ζ with *j* number of proteins and *s* number of bound pZAP70 is shown for the nine FOVs in Supplementary Figure 9 together with the corresponding plots of the inverse of *p*_bound,*j*_ as a function of *j* to derive *K*_d_ from the slope, once the concentration of free pZAP70 is taken into account. Supplementary Table 3 contains the value of *p*_bound,*j*_, for the nine FOVs as well as the difference between this experimental value and the ones derived from a reference with the actual locations of pCD3ζ and pZAP70 molecules placed at random. As expected, practically all actual values of the binding probability are higher than those for the random distribution. The average value of *K*_d_ for pCD3ζ-pZAP70 recovered from the model of the association to clusters, (0.6 ± 0.2) μM, is very similar to the extrapolated value obtained considering the 1:1 interaction. The calculated *K*_d_ value in the micromolar range indicates that pZAP70 has a relatively weaker binding affinity for pCD3ζ chains compared to the stronger nanomolar-range affinity observed for ZAP70 in SPR experiments.^47^ This weaker affinity suggests that pZAP70 dissociates more rapidly from pCD3ζ, supporting a model in which dynamic and transient interactions facilitate efficient signal propagation within the T cell activation pathway. Similarly, recent findings on the dissociation of ZAP70 from pCD3ζ in T cells show that its unbinding can be accelerated by the dephosphorylation of individual phosphotyrosines on CD3ζ, resulting in dissociation constants in the micromolar range.^48^ Such rapid dissociation is likely crucial for allowing swift and reversible signaling events, which are essential for the precise regulation of T cell responses.

In conclusion, we demonstrate the feasibility and strength of our method in accurately determining association and dissociation constants in cellular environments, marking a significant advancement in the SMLM field. Furthermore, we introduce ks-density analysis as a novel tool to evaluate the accessible area for the interacting molecules. This method produces results comparable to those obtained with more traditional techniques, such as Delaunay or Voronoi tessellations, but with significantly reduced sensitivity to parameter values. Ultimately, this allows researchers to implement the developed method without the need to optimize the scale factor. We envision the proposed method to provide a straightforward measurement of association and dissociation for a vast breadth of biological partners, which are already typically visualized via SMLM, thereby expanding the analysis toolkit available to researchers in the field. Looking ahead, as advancements in live-cell SMLM technology continue to improve, our method holds the potential to be adapted for use in live cells, enabling the study of molecular interactions under more physiologically relevant conditions.

## Supporting Information Available

Materials and methods alongside detailed information about the optical setup, cell culture and sample preparation, DNA-PAINT docking and imager sequences, DNA-PAINT imaging experiments and data processing, qPAINT analysis, simulations and data processing, derivation of the equations to show the influence of incomplete labeling-detection on the computation of affinity constants, description of the sequential association model (PDF)
