# Supplementary material for: Quantitative Analysis of Protein–Protein Equilibrium Constants in Cellular Environments Using Single-Molecule Localization Microscopy
Source: Nano Lett. 2024 Oct 21;24(43):13834–42. doi: 10.1021/acs.nanolett.4c04394 (PMC11528428; doi:10.1021/acs.nanolett.4c04394)
Supplement: Supplementary file 1 — nl4c04394_si_001.pdf [file nl4c04394_si_001.pdf]

Quantitative Analysis of Protein-Protein Equilibrium Constants in Cellular Environments using Single-Molecule Localization Microscopy

Luis F. Marcano-García[1], Cecilia Zaza[2], Olivia P. L. Dalby[2,3], Megan D. Joseph[2,3], M. Victoria Cappellari[1†], Sabrina Simoncelli[2,3] and Pedro F. Aramendía,[1*]*

1. Centro de Investigaciones en Bionanociencias -‘‘Elizabeth Jares-Erijman’’ (CIBION), CONICET, Godoy Cruz 2390, 1425 Ciudad de Buenos Aires, Argentina.
2. London Centre for Nanotechnology, University College London, 19 Gordon Street, WC1H 0AH London, United Kingdom.
3. Department of Chemistry, University College London, 20 Gordon Street, WC1H 0AJ London, United Kingdom.

Supplementary Information

Methods

DNA-antibody coupling reactions

An antibody against pCD3ζ (CD247 (pY142), BD Pharmingen) and the Fab Fragment Donkey Anti-Rabbit IgG (Jackson ImmunoResearch, 711-007-003) were conjugated to DNA-PAINT docking strands 5′-Thiol-AACAACAACAACAACAACAA-3′ (Eurofins) and 5′-Thiol-ACACACACACACACACACA-3′ (Eurofins) via maleimide-PEG2-succinimidyl ester coupling reaction, respectively. These sequences feature repetitive (AAC)n or (AC)n to increase the frequency of binding events, which in turn allows the use of relatively low imager strand concentrations without compromising overall imaging times whilst achieving high signal-to-noise ratio and single-molecule localization precision.
1 mM thiolated DNA docking strands were reduced with freshly prepared 250 mM Dithiothreitol (DTT) (Thermo Fisher Scientific) solution for 2h at room temperature. The pCD3ζ antibody was then concentrated using 100kDa Amicon spin filter (Merck/EMD Millipore) before the antibody and Fab were both separately mixed with 20x molar excess of maleimide-PEG2-succinimidyl ester cross-linker (Sigma-Aldrich) for 90 min at 4 °C in the dark. To remove excess DTT and cross-linker, both reactions were purified by spin filtration

using a Microspin Illustra G-25 column (GE Healthcare) and a Zeba spin desalting column (7K MWCO, Thermo Fisher Scientific), respectively. The reduced DNA docking strands were added to the corresponding purified antibody-crosslinker solution at 10x molar excess and incubated on a shaker overnight at 4°C in the dark. Finally, excess DNA was removed from the pCD3ζ and Fab products via 100 kDa and 30 kDa Amicon spin (Merck/EMD Millipore) filtration respectively and stored at 4ºC. Antibody-DNA concentration was measured using a NanoDrop One spectrophotometer (Thermo Fisher Scientific).

Cell culture, activation and immunofluorescence staining of Jurkat T cells

Jurkat E6.1 T cells were cultured in Roswell Park Memorial Institute (RPMI-1640, Thermo Fisher Scientific) medium supplemented with 10% fetal bovine serum (FBS, GIBCO), and 1 mM penicillin and streptomycin (all from Life Technologies). Cells were kept at 37°C and 5% CO_2 in a humid incubator.

To form artificial immunological synapses, a 0.4 mM liposome solution in phosphate buffered saline (PBS) was created with vesicle extrusion through 100 nm polycarbonate filters. The solution consisted of a lipid molar ratio of 97.4 % DOPC (Avanti Polar Lipids, 850375C), 2 % DGS-NTA(Ni) (Avanti Polar Lipids, 790404C), 0.1 % Biotinyl-Cap-PE (Avanti Polar Lipids, 870273C) and 0.5 % PEG5,000-PE (Merck, 880220P-200MG). A glass coverslip was sonicated in 2 % Hellmanex for 30 min, before being washed with ethanol and dried under a flow of nitrogen. The coverslip was plasma cleaned (Diener Zepto plasma cleaner, 40 kHz generator, 90 s at 70 W power) and attached to a six-channel microscopy chamber slide (sticky-Slide VI 0.4, 80608, Ibidi) before adding 50 µL of the 0.4 mM liposome solution for 20 minutes at room temperature to form the lipid bilayer. The bilayer coated chambers were washed 3 x with lipid buffer (2 mM $MgCl_2$, 1 mM $CaCl_2$ and 0.1 % BSA in PBS). 0.1 mM $NiCl_2$ in 2 % BSA in PBS was added to recharge the NTA groups on the lipids and block the bilayer for 20 minutes at room temperature, before washing 3x with lipid buffer. To functionalize the bilayer with biotinylated proteins, 12.5 µg/ml of streptavidin (228-11469-2, Cambridge Bioscience RayBiotech) in lipid buffer was added for 20 min and then washed three times with lipid buffer. For activating T cells, the streptavidin coated bilayer was functionalized with the addition of 3.4 µg/ml biotinylated aCD3 (Biolegend, 317320), 3.4 µg/ml biotinylated aCD28 (Biolegend, 302904) and 200 ng/mL His-tagged iCAM-1 (Thermo Fisher Scientific, A42524) in lipid buffer. Whilst for non-activated conditions only 200 ng/mL His-tagged iCAM-1 alone in lipid buffer was added. After 20 min the bilayer was washed three times with lipid buffer to remove any unbound proteins. The prepared slide was incubated for 15 mins at 37 °C prior to the addition of cells.

T cells were resuspended in PBS at a concentration of $1x10^7$ cells/mL and immediately added to glass chambers coated with functionalized bilayers: glass-supported lipid bilayer (SLB). For activated conditions, the bilayers were functionalized with aCD3, aCD28, and iCAM-1, while for non-activated conditions, only iCAM-1 was used. Following 15 min incubation at 37 °C, cells were fixed for 30 min with a pre-warmed fix solution of 4% paraformaldehyde (LifeTech) at room temperature. Following 3 x washes with 60 mM glycine in PBS, cell membranes were permeabilized for 5 min in 0.1 % Triton X-100 solution (Avantor) and washed with 60 mM glycine in PBS for 3 × 5 min. Cells were then blocked in 5 % BSA for 90 min and subsequently incubated with the different primary and secondary antibodies detailed above.

CD3ζ labeling
Staining of the non-activated T cells was performed with a primary rabbit antibody against CD3ζ (Abcam, ab40804). Staining was done in 5% BSA in PBS at a concentration of 1.7 µg/ml for 1 h at 37°C, then washed with PBS. Secondary staining was performed immediately after with the DNA conjugated anti-rabbit Fab in 5% BSA in PBS at a concentration of 4 µg/ml for 1 h at room temperature. After washing 3x with PBS, 90 nm gold nanoparticles (Cytodiagnostics, G-90-100) were added for 10 minutes for use as fiducial markers. Cells were then washed again 3× in PBS and immediately imaged using the imager strand solutions as described below.

pCD3 and pZAP70 labeling
Staining of the activated T cells was performed with both a primary rabbit antibody against pZAP70 (Cell Signaling, 2704S), and the DNA conjugated anti-pCD3ζ, diluted 1:50 and 1:100, respectively, in 5% BSA for one hour at 37 °C. The cells were then washed 3× 3 min in PBS prior to incubation with the DNA conjugated anti-rabbit Fab fragment as described above. After washing 3x with PBS, 90 nm gold nanoparticles (G-90-100, Cytodiagnostics) were added for 10 minutes for use as fiducial markers. Cells were then washed again in PBS and immediately imaged using the imager strand solutions as described below.

DNA-PAINT Imaging Experiments
Exchange DNA-PAINT imaging was carried out on a custom built total internal reflection fluorescence (TIRF) microscope based on a Nikon Eclipse Ti-2 microscope (Nikon Instruments) equipped with a 100× oil immersion TIRF objective (Apo TIRF, NA 1.49) and a Perfect Focus System. For excitation of Cy3B labeled strands, a 561 nm laser (MPB Communications, 1 W) was used. Laser polarization was adjusted to circular using a polarizer (LPVISC050-MP2, Thorlabs) followed by a quarter waveplate (LAS-051506, Laser 2000). To achieve flat-top TIRF illumination, the beam was expanded up to 6 mm diameter with a variable beam expander (BE02-05-A, Thorlabs) before passing through a beam shaper device (piShaper 6_6_VIS, AdlOptica) that transformed the Gaussian profile of the beam into a collimated flat-top profile. Finally, the beam was focused into the back focal plane of the microscope objective (AC508-300-A-ML, Thorlabs), passed through a clean-up filter (FF01-390/482/563/640-25, Semrock) and coupled into the objective using a beam splitter (Di03-R405/488/561/635-t1-25×36, Semrock). Fluorescence light was spectrally filtered (FF01-446/523/600/677-25, Semrock) and imaged on a sCMOS camera (ORCA-Flash4.0 V3 Digital, Hamamatsu) without further magnification. The final pixel size was of 130 nm in the focal plane, after 2 × 2 binning.

Imager solutions of 3′ terminus Cy3B-labeled DNA imager strands, 0.5 nM of 5′-TGTGTGT-3′, for both CD3ζ and pZAP70 imaging via the DNA conjugated rabbit Fab, and 2 nM of 5′-TTGTTGTT-3′ (Eurofins), for pCD3ζ imaging, were prepared in 0.5 M NaCl and 1 mM EDTA (Invitrogen) in PBS. For fluid exchange the Ibidi µ-Slide VI 0.4 chamber was connected to silicon tubing (Silicon Tubing 0.5 mm ID, Ibidi) via a suitable adaptor (Elbow Luer Connector Male, Ibidi). Each imaging acquisition step was performed by adding the corresponding imager strand solution to the sample followed by a 5 min washing step with 0.5 M NaCl and 1 mM EDTA (Invitrogen) in PBS. Before the next imager strand solution was introduced, we monitored the camera readout to ensure complete exchange of imager solutions. Each imaging step corresponded to sequences of 15,000 frames at 10 Hz acquisition rate and a laser power density of 0.2 kW/cm^2.

DNA-PAINT Image Reconstruction, Cluster Analysis and qPAINT analysis

Images were processed and reconstructed using the Picasso software (Version 0.4.1). Single molecule events were localized from the raw fluorescent DNA-PAINT imaging experiments using the 'Localize' module. Drift correction and multi-color data alignment was performed using a combination of redundant cross-correlation (RCC) and fiducial markers (90 nm gold nanoparticles) approach with the 'Render' module. Localizations with uncertainties greater than 15 nm were removed while no merging was performed for molecules re-appearing in subsequent frames. Super-resolution image rendering was done by plotting each localization as a Gaussian probability distribution with standard deviation equal to its localization precision. Average localization precision of the single molecule localization microscopy experiments was calculated via Nearest-Neighbor (NeNa) analysis[1] within Picasso software[2] (Supplementary Figure 4 and Supplementary Figure 7).

Density-based clustering of whole cell DNA-PAINT datasets was carried out using MATLAB's DBSCAN. In the case of single-labeling DNA-PAINT data (CD3ζ), for 'eps', we utilized a distance of 16 nm, considering a >95 % probability of including a localization with a 6 nm Gaussian dispersion, as determined by NeNa analysis (Supplementary Figure 4). For the case of Exchange DNA-PAINT data, we performed DBSCAN for all single molecule localizations in both channels (pCD3ζ and pZAP70) at the same time, and then divided the clusters per channel. For 'eps', we utilized a distance of 25 nm to account for channel overlap, with the same consideration as above and determined by NeNa analysis (Supplementary Figure 7). For 'minPts', we picked a parameter based on the imager strand's binding frequency and the number of recorded frames; in our case, this value was set at 30 localizations for CD3ζ data, and at 45 localizations for the combined pCD3ζ and pZAP70 data.

For qPAINT and *k*-means analysis we used a custom-written MATLAB (2021a) code. Briefly, qPAINT analysis relies on the pseudo-first order binding kinetics between individual imager and docking strands to determine the number copies of a protein that reside within a cluster of single-molecule localizations. We grouped localizations corresponding to the same cluster and used their timestamps to calculate the sequence of dark times (τ_{OFF}) per cluster. Typical intensity traces can be seen in Figure 3c and Figure 4c inset. The average τ_{OFF} of a cluster of single molecule localizations allows determining how many *k* copies of the protein reside within that cluster of points, by computing $\tau_{OFF,1}/\tau_{OFF}$, where $\tau_{OFF,1}$ denotes the dark time for the case of a single protein. To determine the $\tau_{OFF,1}$ value, we selected small clusters in the CD3ζ (single-labeling); and pCD3ζ and pZAP70 DNA-PAINT data sets, based on their geometrical dimension with maximum point distance of 50 nm. All the dark times per cluster were pooled and used to obtain a normalized cumulative histogram which was then fitted with the following exponential function: $1 - \exp(t/\tau_{OFF})$ to estimate the dark time, τ_{OFF}, per cluster. The inverse of the dark time was calculated for each cluster and stored as the qPAINT index of the cluster (q_i). To obtain the average qPAINT index value for the case of one protein, $q_{i,1}$, we constructed a histogram of the qPAINT indices of small clusters (i.e., clusters with a maximum point distance of 50 nm) and fitted it with a multi-peak Gaussian function. These fits can be seen in the Figure 3c and 4c of the main text. The calibration value obtained with this method was used to estimate the number of CD3ζ for single-labeled experiments; and pZAP70 and pCD3ζ proteins in all the single molecule clusters identified by DBSCAN. After determining the number of *k* copies of the protein that reside in each cluster with qPAINT, we use a distance-based algorithm, known as *k*-means clustering, where *k* corresponds to the

protein copy number per cluster obtained from the qPAINT index. The detailed script to perform such analysis can be found in https://github.com/Simoncelli-lab/qPAINT_pipeline

Nearest neighbor analysis

Nearest neighbor distances (NND) for CD3ζ, and for pCD3ζ and pZAP70 were calculated using the recovered CD3ζ, and pCD3ζ and pZAP70 protein maps, respectively as described above via a custom-written MATLAB routine. To evaluate the significance of the NND distributions, we randomized the positions of CD3ζ for the comparison of CD3ζ – CD3ζ NND distributions, and the positions of one of the two proteins, or both, for the comparison of the NND between pCD3ζ - pZAP70 protein distributions.

Simulations to recover sample's area

To determine the area or volume containing the molecules, we used density-based methods such as Delaunay or Voronoi tessellations and a ks-density based method.

Briefly, in Delaunay tessellation, molecular locations are linked by lines to their nearest neighbors, defining triangles, in a way that the circumference determined by the vertices of any triangle only includes these points and no other point of the distribution. The triangles have decreasing area in higher density regions. A threshold in the area of these triangles (A_T, area of the triangle) can be used as a criterion to determine the area accessible to the particular set of molecules. Voronoi tessellation, on the other hand, divides the surface in polygons containing only one location and including the area closer to this location. As in the Delaunay tessellation, the area of each polygon (A_P, area of the polygon) is smaller, the higher the density. A threshold area can be similarly used to define the accessible area. On the other side, ks-density uses a 2D spread function that replaces each location. The sum of all these point spread functions results in a continuous 2D density function, after normalization. In this procedure, at least one extra parameter is needed to define the point spread function, for example the dispersion, $\sigma_{x,y}$, in case the isotropic Gaussian function is used. Also, in this case, a threshold (*TH*) in the value of the density function is used to define the accessible area.

Simulations to obtain the optimum value of the parameters of the containing area were carried out in self-written routines in Matlab. Irregular surfaces, mimicking cellular forms, were generated. Within this area the same number of molecules of two species, associated with different strength of affinity, were placed (molecular density from 120 to 400 μm^{-2} and an input affinity, as measured by $log\ K_a$, from -3 to 0 in μm^{-2} scale), beginning in each case with one or the other at CSR distribution. The number of associated pairs and isolated molecules were computed according to the chosen value of K_a. Positions were then blurred with a top hat probability function of 10 nm width. The list of localizations was used to generate Delaunay or Voronoi tessellations or the ks-density function.

The implementation of each pattern analysis algorithm, as well as the optimization of their relevant parameters are detailed in the following paragraphs.

For Delaunay and Voronoi tessellations, we determined critical areas for rejection based on μ_{NND} and expansion factors, denoted as F_D or F_V, respectively, which were found by comparing the input and recovered patterns of simulations (see Supplementary Figure 1 and details below). The recovered patterns were formed by combining all triangles with areas $A_T \leq (F_D \cdot \mu_{NND})^2$ for Delaunay or polygons with areas $A_P \leq (F_V \cdot \mu_{NND})^2$ for Voronoi. For ks-density, each molecular location was spread using a 2D Gaussian distribution function of unit area and equal width in the two directions given by $\sigma_{x,y}=F_K \cdot \mu_{NND}$, where F_K is the expansion factor. The threshold (*TH*)

to include localizations was set as 1.5 times the maximum of the Gaussian function for a single molecule. Considering that ks-density distribution function is, in turn, normalized, the threshold value, using this criterion, is $TH = 1.5 / (\sqrt{2\pi}\cdot\sigma_{x,y}\cdot N_T)$, where N_T is the total number of localizations of the ensemble, making ks-density a one-parameter model.

To determine the best value of the expansion factor for each of the methods, we explored an interval of F_D, F_V, and F_K between 1 and 4, in simulations covering the density and *log* K_a ranges mentioned above. To evaluate the goodness of the fit, we classified each pixel of the recovered pattern in comparison with the input according to the confusion matrix, where TP corresponds to the true positive pixels (coincident in both cases); FP represents the false positive pixels (belonging to the recovered pattern but not to the original); FN denotes the false negative pixels (the opposite of FP); TN represents the case of the true negative pixels (absent in both patterns). To choose the optimization parameter, we focused our attention on the balance of correct pixels (TP) compared to total erroneous ones (FP + FN), while disregarding TN pixels because they do not carry any information. These considerations point to F1-score, which is defined by F1 = 2·TP / (2·TP + FP + FN), as the most suitable optimization criterion. A unity F1-score means that the original pattern is exactly recovered. Supplementary Figure 2 and Supplementary Table 1 display F1-score for an average of ten simulations under each density. The maximum of F1 can be used to determine the most suitable parameters for each pattern analysis: F_D, F_V, and F_K.

The results are summarized on Supplementary Table 1, from which the suitable value of F_V, F_D or F_K can be chosen to determine the containing area by the respective method. It is worth noting that at least one of the species must be at pseudo random distribution to correctly use this table. In case of highly clustered distributions, the center of mass of the clusters, and its corresponding μ_{NND}, can be tested to be pseudo-randomly distributed and thus used to obtain the expansion factor from Supplementary Table 1. Using the locations within the recovered area, pairs and isolated molecules were identified and used to compute K_a.

Proximity limit criterion and evaluation of K_a.

To establish a proximity limit to distinguish associated from isolated molecules, we compared, as a function of the limit distance for affinity, the computed value of K_a of the simulation with the input value of this parameter. Ideally, the two should coincide. We have to consider also that it is not reasonable to choose a proximity limit smaller than the expected uncertainty in the distance between two localizations, which we can estimate as the average uncertainty in each localization (5 nm in the simulations) multiplied by $(2)^{1/2}$. Supplementary Figure 3 shows the difference between computed and input *log* K_a. Between 10 and 15 nm, this difference is within ±0.3 logarithmic units, being smaller at 15 nm for the higher values of affinity. For this reason, we considered 15 nm as the proximity limit to compute all values of K_a in the simulations. Second, a reciprocity criterion between the localizations was considered, i.e., only if the first nearest neighbor within the proximity distance of localization A is a localization B and the first nearest neighbor of this localization B is the same localization A, were they considered as an associated pair. In the end, we could calculate $-\Delta G°/RT = \log K_a$ and compare it to the input. An average of ten simulations under the same conditions were performed for each density and affinity.

In all cases, the computed value for the affinity constant was compared to the one derived from CSR of the two partners at the same total density in the same environment. In this scenario, we

tested the three possibilities: either of the two components in its experimental distribution and the other in CSR or both in CSR. For the Langmuir type association, the experimental distribution of pCD3ζ was used and pZAP70 was placed at CSR. To demonstrate that this later criterion does not introduce any bias in the selection of pCD3ζ locations, we compared the distributions of pCD3ζ obtained by determining the containing area using the center of mass of the clusters, with the one obtained by using the locations of both partners together. The correlation of the locations through the plot of the cumulative complementary distribution function of the first nearest neighbor of the cross correlation shows that more than 99% of the locations coincide in the two distributions within less that 1 nm (data not shown).

Supplementary Figures

Density (μm^{-2})	Log K_a	Method		
		**Delaunay**	**Voronoi**	**ks-density**
120	-3	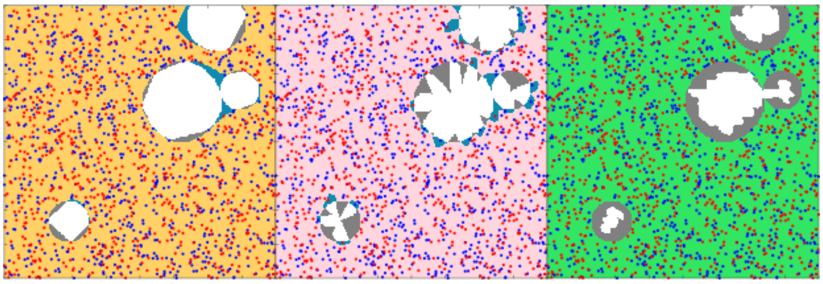		
	0	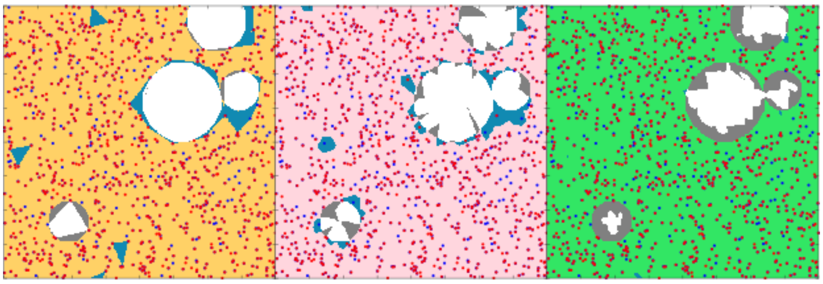		
400	-3	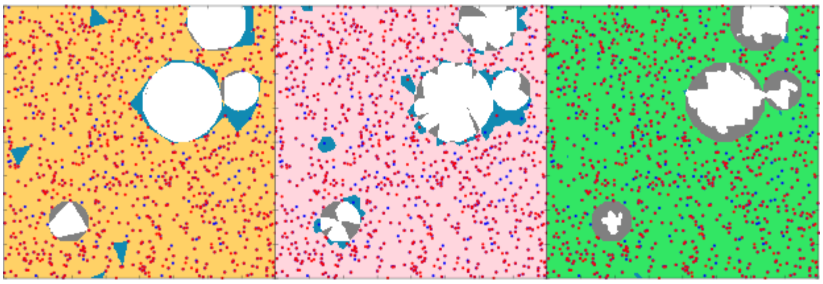		
	0	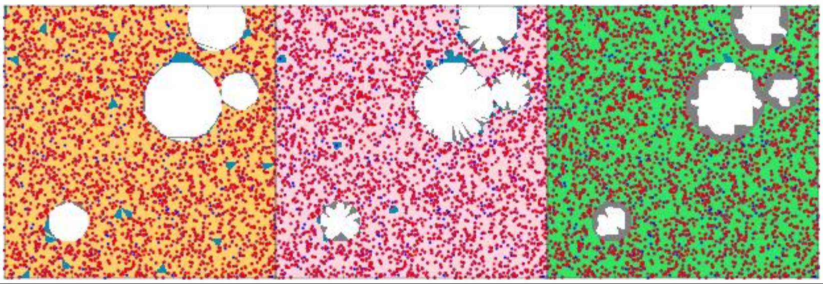		

Supplementary Figure 1. Recovered patterns computed from 2D simulations of interacting species at different molecular density and association affinity. Recovered patterns with the respective expansion factor are compared to the original one (FP: grey, FN: sky-blue, TN: white and TP: yellow, pink and green to Delaunay, Voronoi tessellation and ks-density methods, respectively). Displayed regions are 4x4 μm^2.

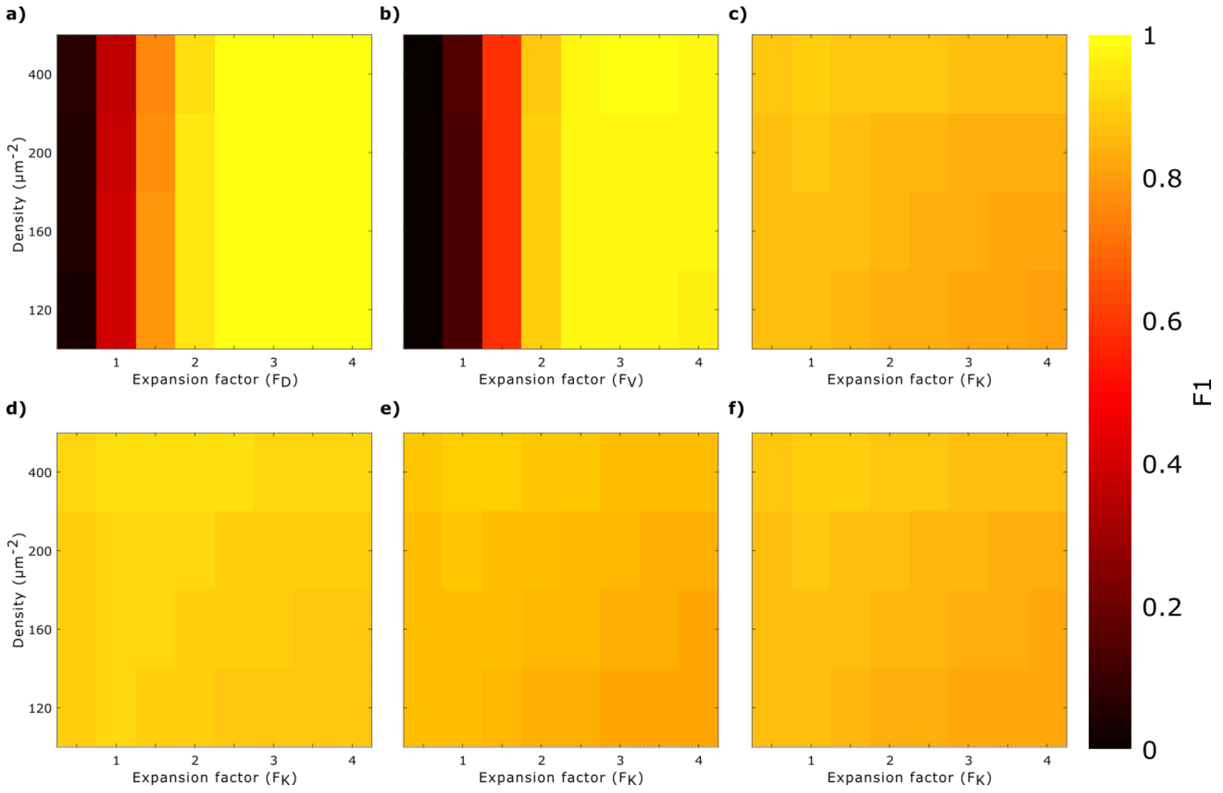

Supplementary Figure 2. F1-Score computed as a function of the expansion factor used to recover the simulated pattern. Simulations were performed using *log* K_a = -2. To compute the recovered patterns **(a)** F_D was used in the Delaunay tessellation method; **(b)** F_V was used in the Voronoi tessellation method; and **(c)** – **(f)** F_K was used in the ks-density method with different TH values given by TH = $x / (\sqrt{2\pi} \cdot \sigma_{x,y} N_T)$: **(c)** x = 1; **(d)** x = 1.5; **(e)** x = 2.0; **(f)** x = 2.5. F1 score was computed as described in the main text, such that a unity F1-score means that the original pattern is exactly recovered.

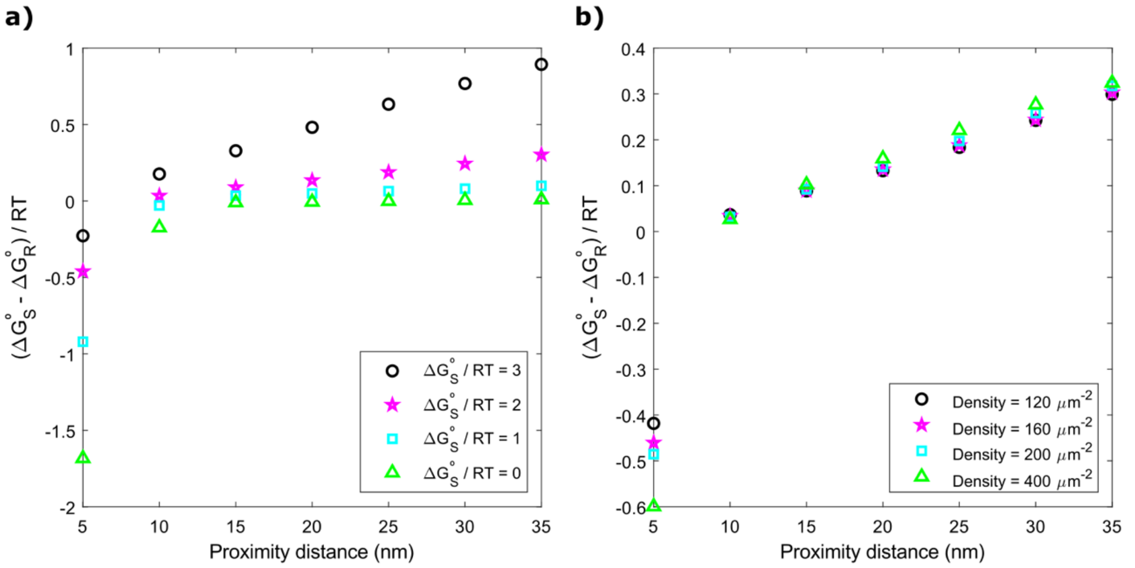

Supplementary Figure 3: In simulated distributions, difference between computed log *K_a* and the input value (a) for different values of this parameter, as indicated, and a total molecular density of 160 µm^{-2}. **(b)** for different molecular density, as indicated, and input log K_a = -2.

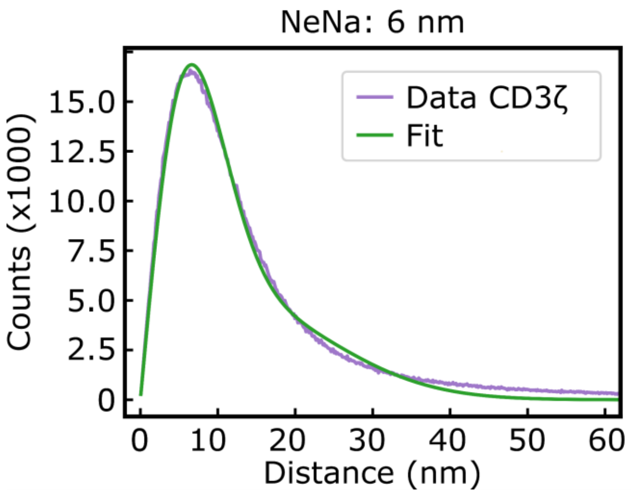

Supplementary Figure 4. Overall localization precision of all super-resolution DNA-PAINT images of CD3ζ based on Nearest-Neighbor analysis (NeNa).

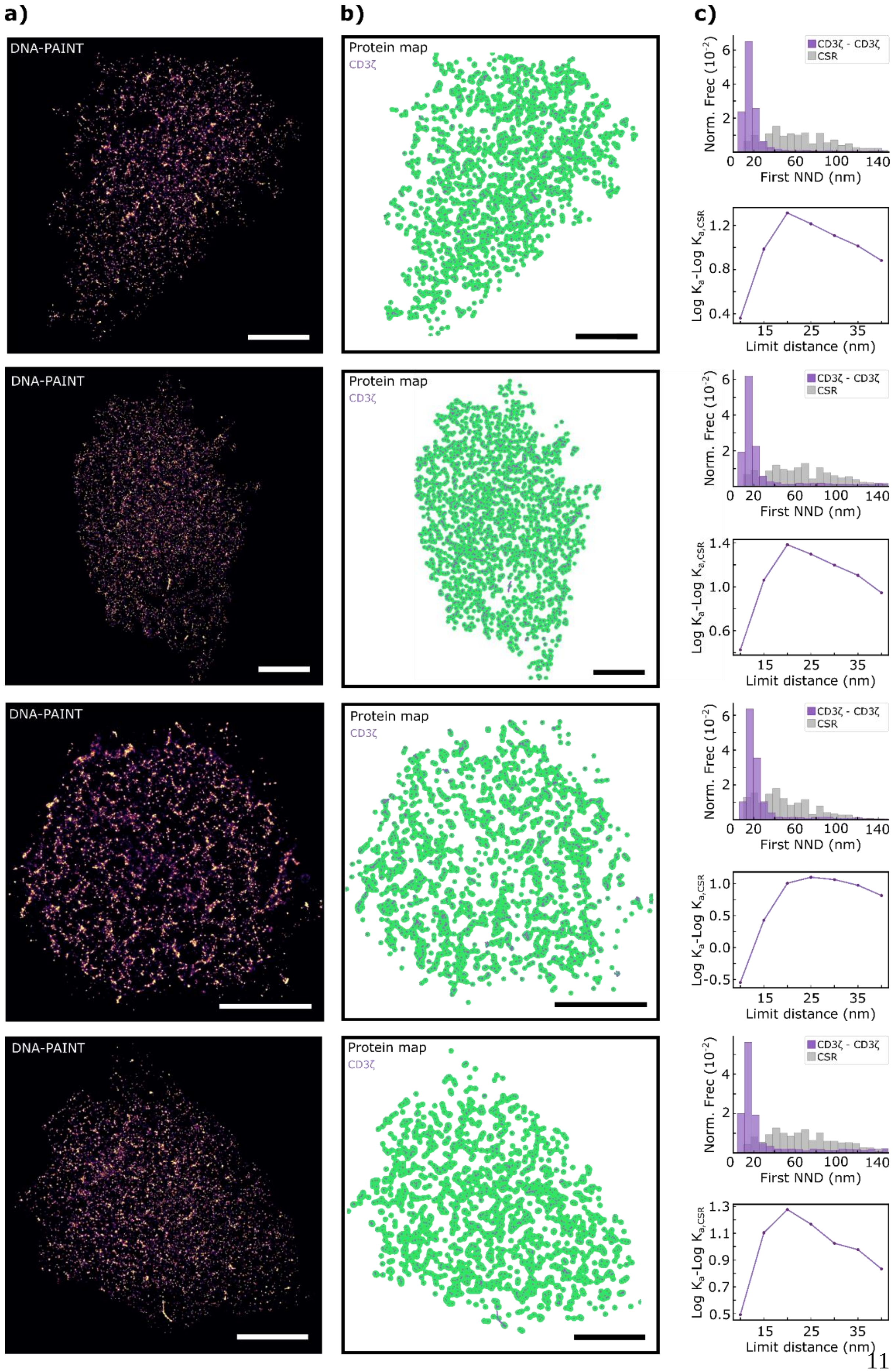

a)
b)
c)
DNA-PAINT
Protein map
CD3ζ
CD3ζ - CD3ζ
CSR
Norm. Frec (10^{-2})
First NND (nm)
Log K_a-Log $K_{a,CSR}$
Limit distance (nm)

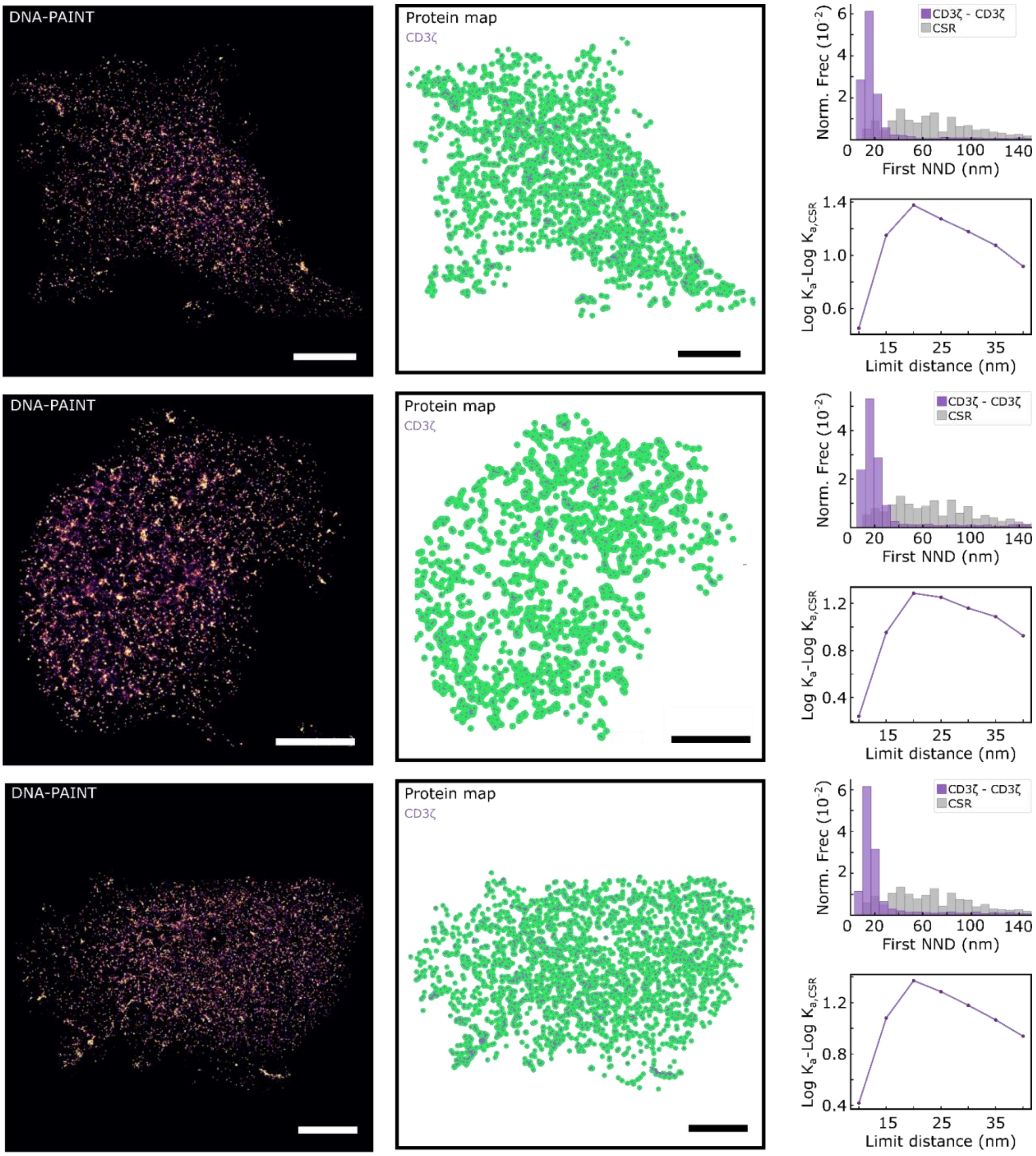

Supplementary Figure 5. Comprehensive data set for the association analysis of the T-cell receptors. **(a)** Super-resolution DNA-PAINT image of CD3ζ in resting (non-activated) Jurkat T-cell. **(b)** Surface area (green) of Jurkat T-cell CD3ζ distribution (purple) as calculated by ks-density. **(c) top:** Whole-cell analysis of first nearest neighbor distances (NNDs) of CD3ζ.

The histograms of NNDs for the case of complete spatial randomness (CSR) are represented in grey. **Bottom:** Computed *log* K_a as a function of the proximity limit threshold utilized to consider proteins as associated pairs. Scale bar represents 3 μm. The association analysis for cell 1 is shown separately in Figure 3.

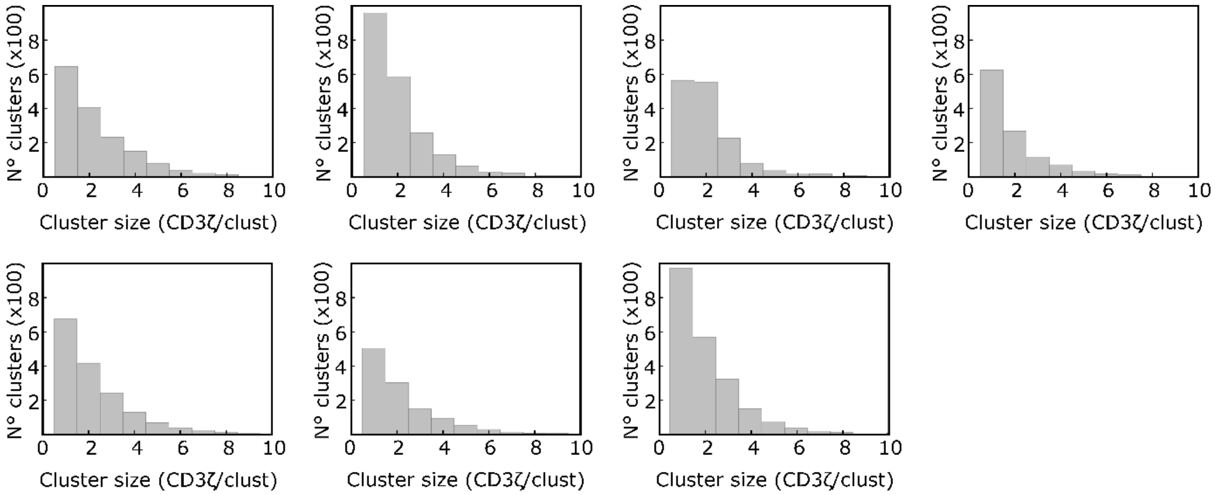

Supplementary Figure 6. Distribution of cluster size of CD3ζ for the entire dataset analyzed (cell 2 to cell 8). The distribution for cell 1 is shown separately in Figure 3d.

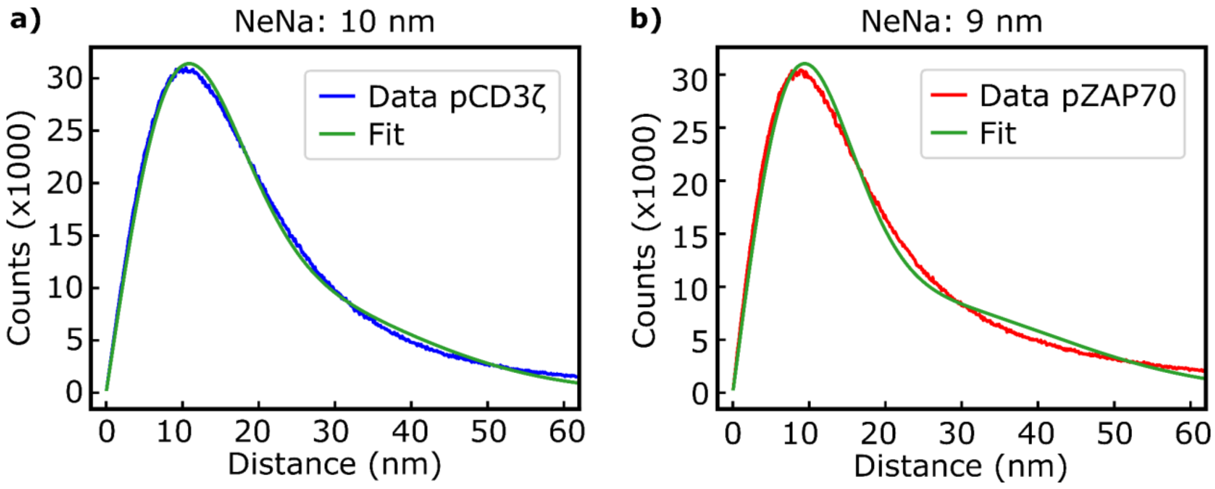

Supplementary Figure 7. Overall localization precision of all super-resolution DNA-PAINT images of pCD3ζ and pZAP70 based on Nearest- Neighbor analysis (NeNa).

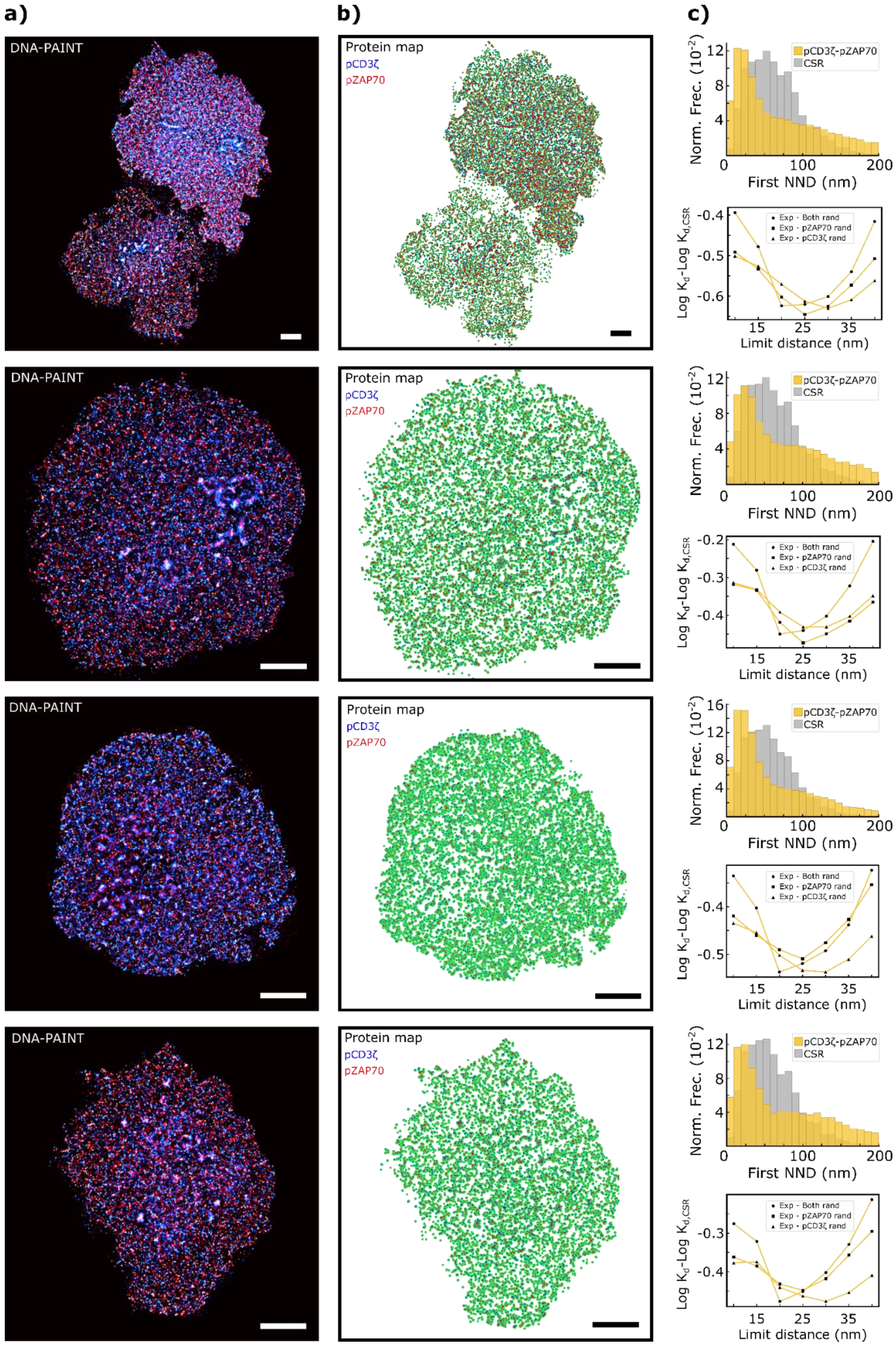

a)
b)
c)
DNA-PAINT
Protein map
pCD3ζ
pZAP70
pCD3ζ-pZAP70
CSR
Norm. Frec. (10^-2)
First NND (nm)
Exp - Both rand
Exp - pZAP70 rand
Exp - pCD3ζ rand
Log K_d-Log K_d,CSR
Limit distance (nm)

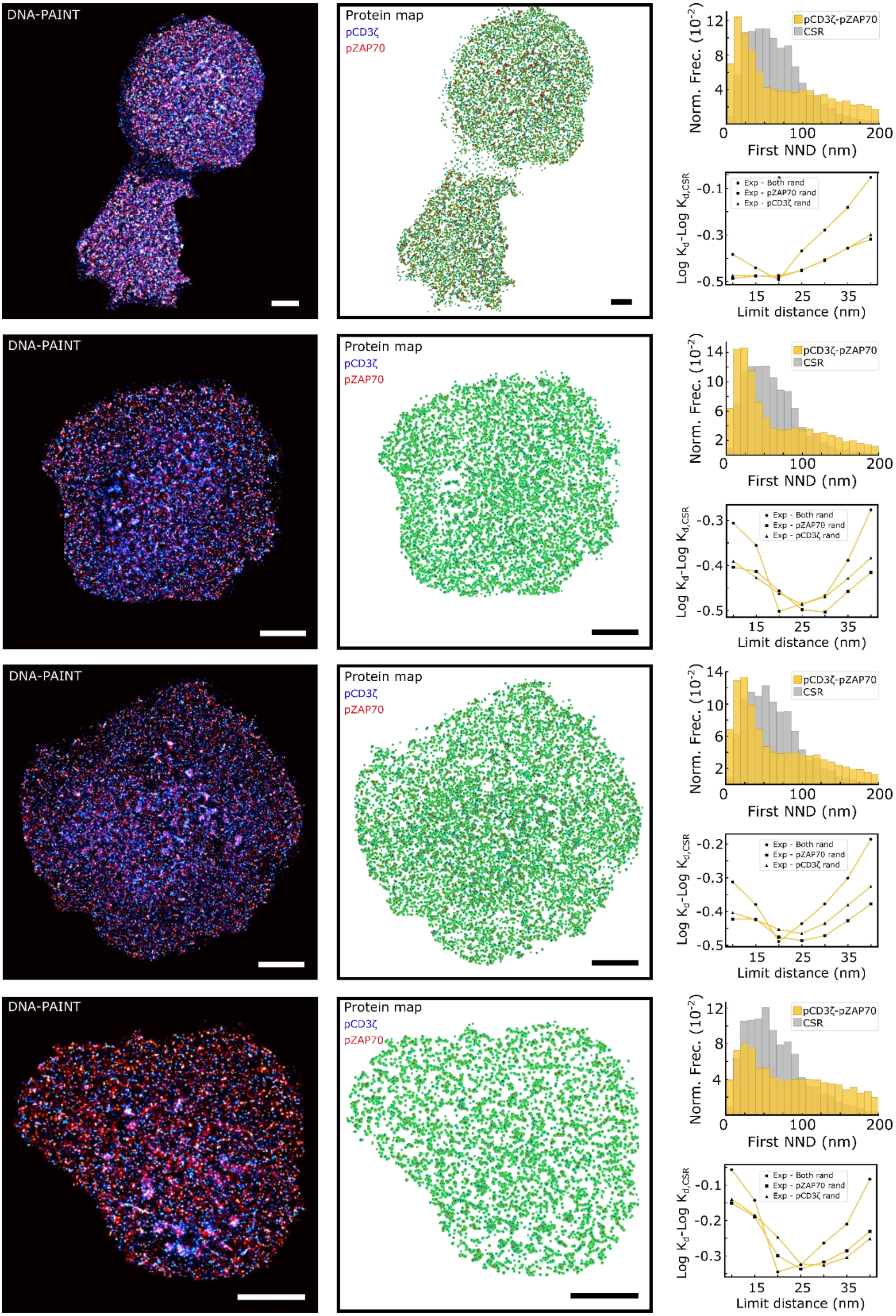

DNA-PAINT
Protein map
pCD3ζ
pZAP70
pCD3ζ-pZAP70
CSR
Norm. Frec. (10^{-2})
First NND (nm)
Exp - Both rand
Exp - pZAP70 rand
Exp - pCD3ζ rand
Log K_d-Log $K_{d,CSR}$
Limit distance (nm)

Supplementary Figure 8. Comprehensive data set for the dissociation analysis of the T-cell signaling proteins pCD3ζ and pZAP70. **(a)** Super-resolution DNA-PAINT image of pCD3ζ (blue) and pZAP70 (red) proteins in activated Jurkat T-cell. **(b)** Surface area (green) of Jurkat T-cell pCD3ζ (blue) and pZAP70 (red) distribution as calculated by ks-density. **(c) top:** Whole-cell analysis of first nearest neighbor distances (NNDs) of pCD3ζ-pZAP70. The histograms of NNDs for the case of complete spatial randomness (CSR) are represented in grey. **Bottom:** Computed $log\ K_d$ as a function of the proximity limit threshold utilized to consider proteins as associated pairs. CSR was computed considering three possibilities, either of the two components in its experimental distribution and the other in CSR or both in CSR. Scale bar represents 3 μm. The dissociation analysis for cell 1 is shown separately in Figure 4.

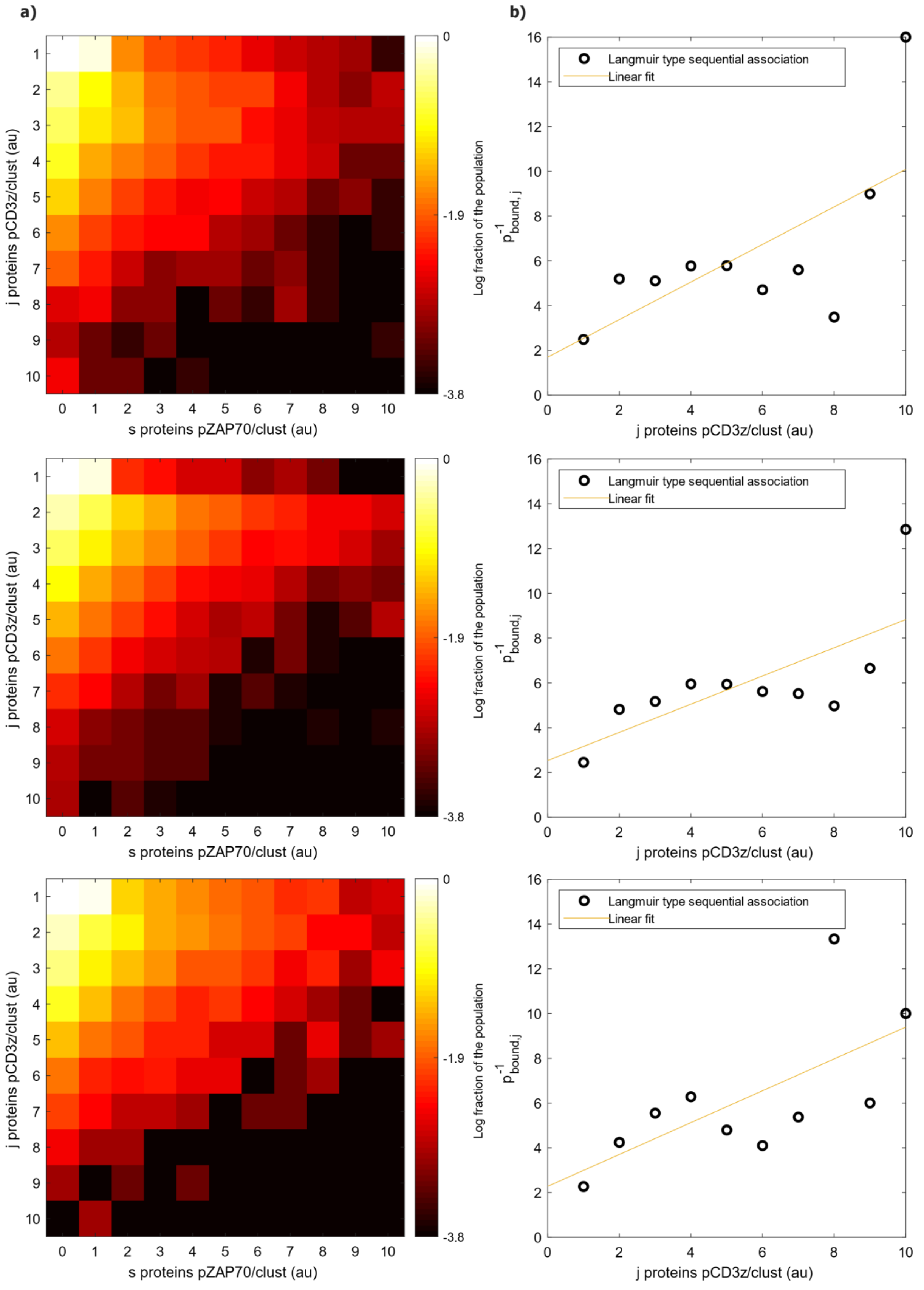
a)
b)
j proteins pCD3z/clust (au)
s proteins pZAP70/clust (au)
Log fraction of the population
0
-1.9
-3.8
Langmuir type sequential association
Linear fit
$p^{-1}_{bound,\,j}$
j proteins pCD3z/clust (au)

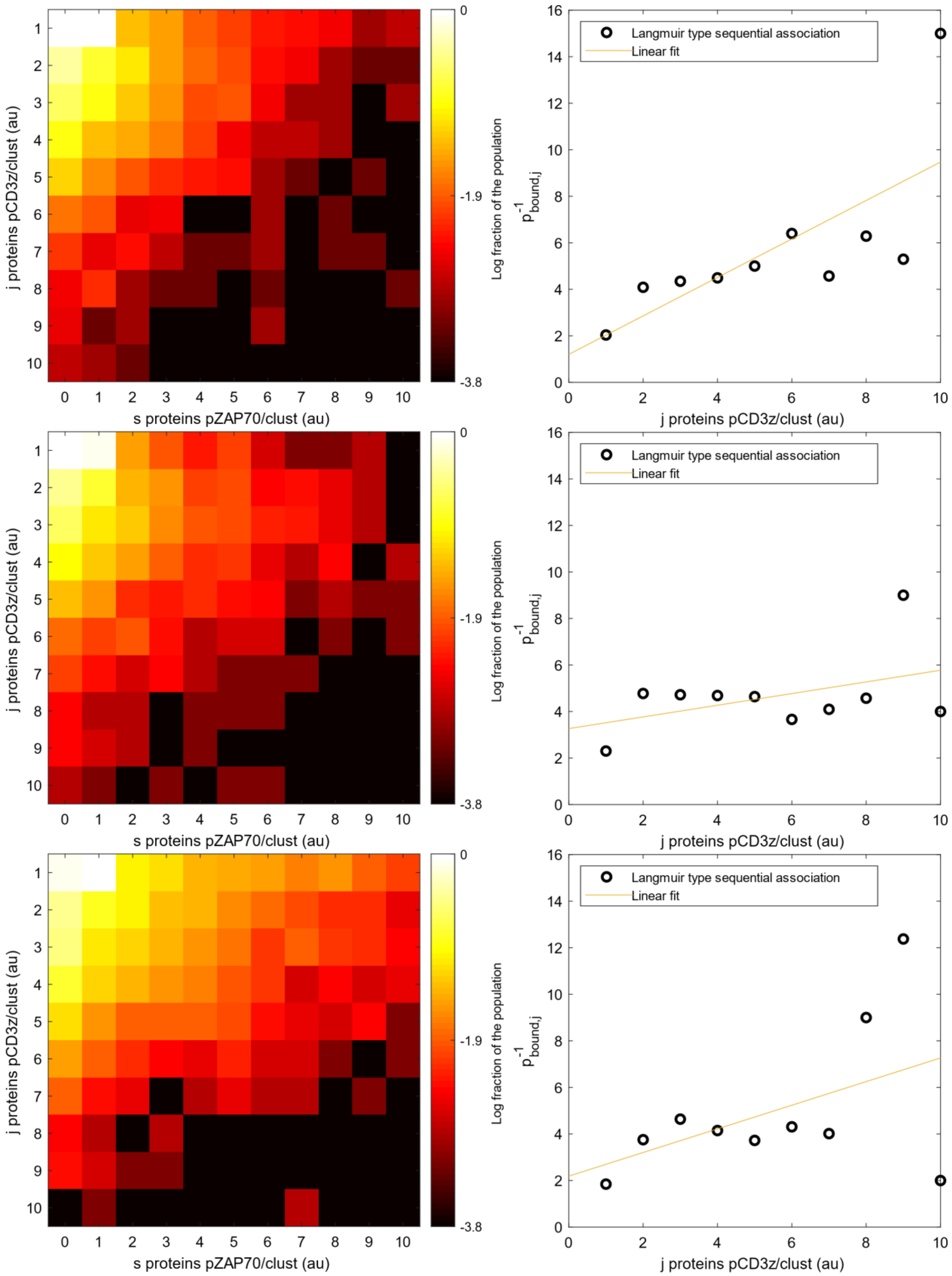

j proteins pCD3z/clust (au)
s proteins pZAP70/clust (au)
Log fraction of the population
0
-1.9
-3.8
$p^{-1}_{bound,j}$
Langmuir type sequential association
Linear fit
j proteins pCD3z/clust (au)

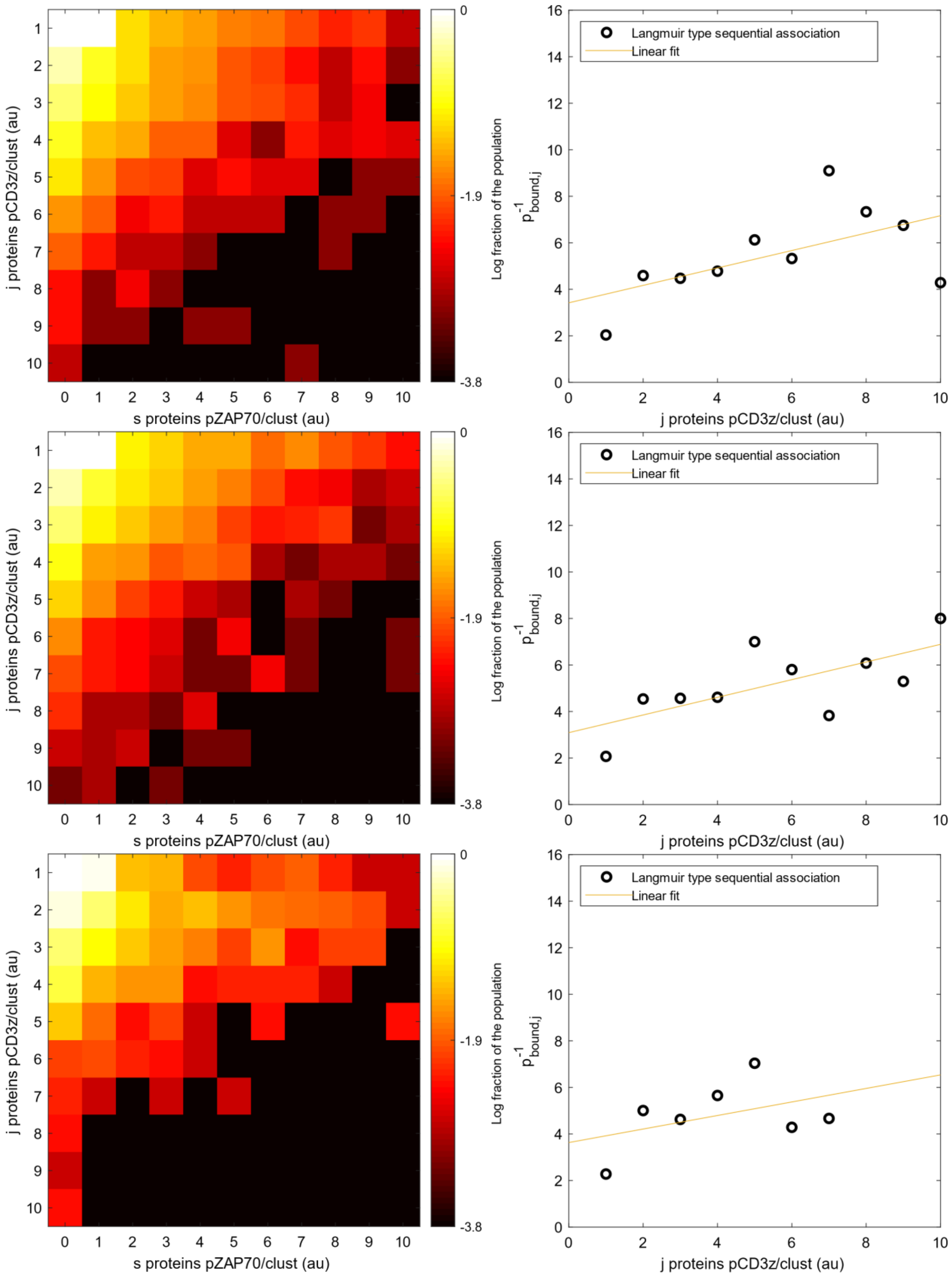

Supplementary Figure 9. Comprehensive data set for Langmuir type sequential association analysis of the T-cell signaling proteins pCD3ζ and pZAP70. (a) Fraction of population (in

logarithmic scale) in whole cells of pCD3ζ clusters with j units and s proteins pZAP70 in activated Jurkat T-cell. (b) Plot of inverses of probability of being bound as a function of j units of pCD3ζ.

Supplementary Tables

Supplementary Table 1: Optimization of parameters with maximum values of F1 computed for each method for simulations of $log\ K_a$ = -2.

**Density (µm^{-2})**				**8**	**10**	**40**	**80**	**120**	**160**	**200**	**400**	**800**
**Delaunay tessellation**			**Max F1**	0.96	0.96	0.98	0.99	0.99	0.99	0.99	1.00	1.00
			**F_D**	2.5	2.5	2.5	3.0	3.0	3.0	3.0	3.5	3.5
**Voronoi tessellation**			**Max F1**	0.94	0.94	0.96	0.97	0.98	0.98	0.98	0.99	0.99
			**F_V**	3.0	3.0	3.0	3.0	3.0	3.0	3.0	3.0	3.0
**ks-density**	**TH** $(x/(\sqrt{2\pi}\cdot\sigma_{x,y}\cdot N_T)$	**1**	**Max F1**	0.75	0.76	0.82	0.85	0.86	0.87	0.88	0.90	0.91
			**F_K**	0.5	0.5	0.5	0.5	1.0	1.0	1.0	1.0	1.0
		**1.5**	**Max F1**	0.75	0.76	0.82	0.85	0.86	0.87	0.88	0.90	0.91
			**F_K**	0.5	0.5	0.5	0.5	1.0	1.0	1.0	1.0	1.0
		**2.0**	**Max F1**	0.75	0.76	0.82	0.85	0.86	0.87	0.88	0.90	0.91
			**F_K**	0.5	0.5	0.5	1.0	1.0	1.0	1.0	1.0	1.0
		**2.5**	**Max F1**	0.75	0.76	0.82	0.85	0.86	0.87	0.88	0.90	0.92
			**F_K**	0.5	0.5	0.5	1.0	1.0	1.0	1.0	1.0	1.0
		**3.0**	**Max F1**	0.75	0.76	0.82	0.85	0.86	0.87	0.88	0.90	0.92
			**F_K**	0.5	0.5	0.5	1.0	1.0	1.0	1.0	1.0	1.0

Supplementary Table 2: Values of the equilibrium constant of the stepwise formation of clusters of j number of CD3ζ proteins from the association of a monomer to a cluster of j-1 proteins. Values are the average for the 8 cells shown in Supplementary Figure 5.

j-mer	- (Log Ka ± std)<br>Scale μm^{-2}	Ka<br>[× 10^{-3} μm^2]
**2**	1.3 ± 0.1	52
**3**	1.4 ± 0.2	42
**4**	1.4 ± 0.2	43
**5**	1.4 ± 0.1	41
**6**	1.4 ± 0.1	42
**7**	1.3 ± 0.2	46
**8**	1.5 ± 0.3	35

Supplementary Table 3. Langmuir model. Binding probability of pZAP70 to each type of pCD3ζ clusters for 9 FOV in whole cells (Table 3A) and its difference compared to a random distribution of pZAP70 (Table 3B). Green shaded: Probability greater than random. Brown shaded: Probability equal to random. Pink shaded: Probability smaller than random.

Table 3A:

		Success probability p								
		**FOV1**	**FOV2**	**FOV3**	**FOV4**	**FOV5**	**FOV6**	**FOV7**	**FOV8**	**FOV9**
**Clusters with j units of pCD3ζ**	**1**	0.40	0.41	0.44	0.49	0.43	0.54	0.49	0.48	0.44
	**2**	0.19	0.21	0.24	0.24	0.21	0.27	0.22	0.22	0.20
	**3**	0.20	0.19	0.18	0.23	0.21	0.22	0.22	0.22	0.22
	**4**	0.17	0.17	0.16	0.22	0.21	0.24	0.21	0.22	0.18
	**5**	0.17	0.17	0.21	0.20	0.22	0.27	0.16	0.14	0.14
	**6**	0.21	0.18	0.24	0.16	0.27	0.23	0.19	0.17	0.23
	**7**	0.18	0.18	0.19	0.22	0.24	0.25	0.11	0.26	0.21
	**8**	0.29	0.20	0.08	0.16	0.22	0.11	0.14	0.16	0
	**9**	0.11	0.15	0.17	0.19	0.11	0.08	0.15	0.19	0
	**10**	0.06	0.08	0.10	0.07	0.25	0.50	0.23	0.12	0

Table 3B:

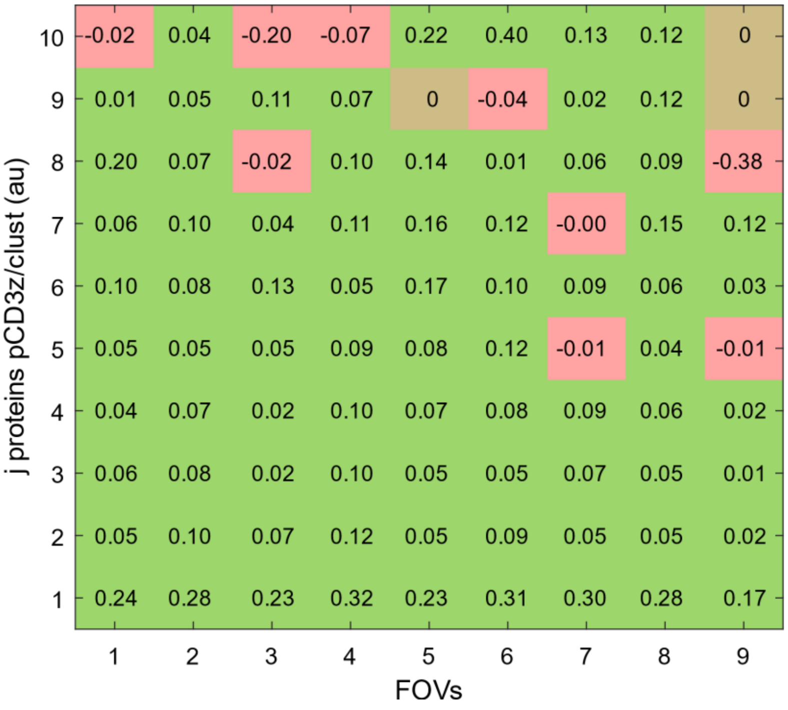

Supplementary Notes

Supplementary Note 1

Effect of incomplete labeling-detection efficiency in the value of K_a or K_d

This note is a summary of the analysis developed in reference: *Photochemical and Photobiological Sciences* **21**, 1751–1760 (2022) to extrapolate the actual value of a 1:1 association constant by taking quantitatively into account the effect of detecting only a fraction, f_A and f_B, of the total number fluorophores of each associated partner. These factors include the added effects of incomplete labeling-detection of the fluorophores. The underlying assumption is that this detection factor depends on the fluorophore and not on the aggregation state of the protein partners.

We begin by expressing the total number of molecules of each species, namely A, B, or AB, depending on its detection state:

$$N_A = f_A . N_A + (1 - f_A). N_A \qquad \text{Eq. S1}$$

$$N_B = f_B . N_B + (1 - f_B). N_B \qquad \text{Eq. S2}$$

$$N_{AB} = f_A . f_B . N_{AB} + f_A . (1 - f_B). N_{AB} + (1 - f_A). f_B . N_{AB} + (1 - f_A). (1 - f_B). N_{AB} \qquad \text{Eq. S3}$$

In Eqs. S1-2, in the second member, the terms represent the number of detected and undetected molecules, respectively. In Eq S3, the terms in the second member represent the number of detected aggregates, the number of aggregates counted as A, B or undetected, respectively.

Under these considerations, the number of molecules identified as belonging to each species (identified with the superscript D) are:

$$N_A{}^D = f_A . N_A + f_A . (1 - f_B). N_{AB} \qquad \text{Eq. S4}$$

$$N_B{}^D = f_B . N_B + (1 - f_A). f_B . N_{AB} \qquad \text{Eq. S5}$$

$$N_{AB}{}^D = f_A . f_B . N_{AB} \qquad \text{Eq. S6}$$

These expressions for the detected molecules belonging to each species are used to compute the experimental value $Q_{AB}^{exp} = \frac{N_{AB}^D}{N_A^D . N_B^D}$:

$$\frac{1}{Q_{AB}^{exp}} = \frac{(f_A . N_A + f_A . (1 - f_B). N_{AB}).(f_B . N_B + (1 - f_A). f_B . N_{AB})}{f_A . f_B . N_{AB}} \qquad \text{Eq. S7}$$

After rearrangement, Eq. S7 can be written as:

$$\frac{1}{Q_{AB}^{exp}} = \frac{1}{Q_{AB}} + (1 - f_A). N_A + (1 - f_B). N_B + (1 - f_A)(1 - f_B). N_{AB} \qquad \text{Eq. S8}$$

The values reported in the work correspond to:

$$log(K_a) = log\left(Q_{AB}^{exp}.S\right) \quad \text{Eq. S9}$$

$$log\left(K_{a,extr}\right) = log(Q_{AB}.S) \quad \text{Eq. S10}$$

With S, the surface containing the proteins in µm^2. When needed, the volume can be determined by multiplying the surface containing by the TIRF depth.

Eq. S8 demonstrates that the actual value of Q_{AB} can be obtained by extrapolation of the experimental values to infinite dilution.

Supplementary Note 2:

Sequential association

The association scenario describes a sequential clustering of pCD3ζ molecules and a Langmuir type of occupation of ZAP70 to each cluster[3]. At equilibrium, the simplest model to describe the clustering of pCD3ζ assumes that the diffusion of the monomer is independent of cluster size, whereas the dissociation rate of one monomer of the cluster is directly proportional to their number. Under these considerations, the probability of finding a cluster with j elements of pCD3ζ will display a Poisson statistic.

$$p_j = \frac{\langle n_A \rangle^j}{j!} \cdot e^{-\langle n_A \rangle} \quad \text{Eq. S11}$$

While the equilibrium constant for each step should be:

$$K_{a,j} = \frac{k_e}{j \cdot k_d} \quad \text{Eq. S12}$$

where k_e is the diffusion rate constant of the monomer to the clusters and k_d is the unimolecular rate constant of the dissociation of one monomer.

Furthermore, if we assume that pZAP70 dissociates 1:1 from any pCD3ζ in a cluster with equal probability, independent of the presence of other pZAP70 molecules in it and of the cluster size, the number of pZAP70 molecules within clusters of equal number of pCD3ζ molecules, follows a binomial distribution and a Langmuir type occupancy as a function of free pZAP70 concentration at equilibrium. In all equations that follow, A represents pCD3ζ and B, pZAP70.

Each pCD3ζ molecule in this cluster can be bound to a pZAP70 molecule with a probability of being bound or free:

$$p_{bound,j} = \frac{\left(\frac{k_b}{j}.k_u\right).[B]}{1+\left(\frac{k_b}{j}.k_u\right).[B]} \qquad p_{free,j} = 1 - p_{bound,j} \quad \text{Eqs. S13a, S13b}$$

where k_b is the total binding rate constant for the cluster and k_u is the first order rate constant for unbinding for one site. Both are assumed equal for all clusters. In this model:

$$K_d = \frac{k_u}{k_b} \quad \text{Eq. S14}$$

The probability of finding s pZAP70 molecules in a cluster of j pCD3ζ molecules is:

$$p_s = \frac{j!}{s!.(j-s)!}.\left(\frac{\left(\frac{k_b}{j}.k_u\right).[B]}{1+\left(\frac{k_b}{j}.k_u\right).[B]}\right)^s \cong . \left(\frac{1}{1+\left(\frac{k_b}{j}.k_u\right).[B]}\right)^{(j-s)} = \frac{j!}{s!(j-s)!}.\left(p_{bound,j}\right)^s.\left(1-p_{bound,j}\right)^{j-s}$$

Eq. S15

Finally, the probability of having a cluster with j pCD3ζ molecules and s number of bound pZAP70 molecules is the combination of the two statistics described:

$$P_{j,s} = \frac{<n_A>^j}{j!}.\exp(-<n_A>).\frac{j!}{s!(j-s)!}.\left(p_{bound,j}\right)^s.\left(1-p_{bound,j}\right)^{j-s}$$ Eq. S16

By fitting the distribution of pZAP70 molecules in clusters of different types in one image, $p_{bound,j}$ can be obtained as a function of j. from Eq. S13a, a plot of inverses of $p_{bound,j}$ vs. j renders $(K_d)^{-1}$ from the slope, once [B] is known.

Supplementary Note 3

pZAP70 bound to different ITAM sites

We analyze the scenario in which pZAP70 is bound to an ITAM site different from the ζ end (pY142). In this case, the image detects proximity, and it is therefore computed as an associated pair. We can quantitatively evaluate this situation if we consider a number k of binding sites, S_i, with i = 1,..,k, each with affinity K_i for pZAP70 in the ITAM unit. At equilibrium:

$$K_i = \frac{[S_i A]}{[S_i].[A]}$$ Eq. S17

We point out that we are computing an effective constant, that can be expressed as:

$$K_e = \frac{\sum_{i=1}^{k}[S_i A]}{[A].\sum_{i=1}^{k}[S_i]}$$ Eq. S18

The sum of all sites with bound pZAP70 is:

$$\sum_{i=1}^{k}[S_i A] = [A].\sum_{i=1}^{k} K_i[S_i]$$ Eq. S19

If we replace this later equation in Eq. S18 for K_e

$$K_e = \frac{\sum_{i=1}^{k} K_i[S_i]}{\sum_{i=1}^{k}[S_i]} = \sum_{i=1}^{k}\left(K_i.\frac{[S_i]}{\sum_{j=1}^{k}[S_j]}\right)$$ Eq. S20

Which can be interpreted as the weighted average of all the binding constants. The dissociation constant, informed in the work, is the inverse of this average K_e.

Supplementary References

(1) Endesfelder, U.; Malkusch, S.; Fricke, F.; Heilemann, M. A Simple Method to Estimate the Average Localization Precision of a Single-Molecule Localization Microscopy Experiment. *Histochem Cell Biol* **2014**, *141* (6), 629–638. https://doi.org/10.1007/s00418-014-1192-3.

(2) Schnitzbauer, J.; Strauss, M. T.; Schlichthaerle, T.; Schueder, F.; Jungmann, R. Super-Resolution Microscopy with DNA-PAINT. *Nat Protoc* **2017**, *12* (6), 1198–1228. https://doi.org/10.1038/nprot.2017.024.

(3) Taylor, M. J.; Husain, K.; Gartner, Z. J.; Mayor, S.; Vale, R. D. A DNA-Based T Cell Receptor Reveals a Role for Receptor Clustering in Ligand Discrimination. *Cell* **2017**, *169* (1), 108-119.e20. https://doi.org/10.1016/J.CELL.2017.03.006.
